# Inhibition of Interleukin-33 to Reduce Glomerular Endothelial Inflammation in Diabetic Kidney Disease

**DOI:** 10.1016/j.ekir.2024.03.009

**Published:** 2024-03-18

**Authors:** Alexis Hofherr, Elena Liarte Marin, Barbara Musial, Asha Seth, Tim Slidel, James Conway, David Baker, Pernille B.L. Hansen, Benjamin Challis, Stefano Bartesaghi, Maria Bhat, Roberto Pecoits-Filho, Xiao Tu, Viknesh Selvarajah, Kevin Woollard, Hiddo J.L. Heerspink

**Affiliations:** 1 Research and Early Clinical Development, Cardiovascular, Renal and Metabolism, BioPharmaceuticals R&D, AstraZeneca, Gothenburg, Sweden; 2 Bioscience Renal, Research and Early Development, Cardiovascular, Renal and Metabolism, BioPharmaceuticals R&D, AstraZeneca, Cambridge, UK; 3 Bioinformatics, Oncology R&D, AstraZeneca, Cambridge, UK; 4 Bioinformatics, Oncology R&D, AstraZeneca, Gaithersburg, Maryland, USA; 5 Bioscience Metabolism, Research and Early Development, Cardiovascular, Renal and Metabolism, BioPharmaceuticals R&D, AstraZeneca, Cambridge, UK; 6 Translational Science and Experimental Medicine, Research and Early Clinical Development, Cardiovascular, Renal and Metabolism, BioPharmaceuticals R&D, AstraZeneca, Gothenburg, Sweden; 7 Arbor Research Collaborative for Health, Ann Arbor, Michigan, USA; 8 School of Medicine, Pontificia Universidade de Catolica do Parana, Curitiba, Brazil; 9 The George Institute for Global Health, University of New South Wales Sydney, Sydney, New South Wales, Australia; 10 Research and Early Clinical Development, Cardiovascular, Renal and Metabolism, BioPharmaceuticals R&D, AstraZeneca, Gaithersburg, Maryland, USA; 11 Research and Early Clinical Development, Cardiovascular, Renal and Metabolism, BioPharmaceuticals R&D, AstraZeneca, Cambridge, UK; 12 Department of Clinical Pharmacy and Pharmacology, University of Groningen, University Medical Center Groningen, Groningen, The Netherlands

**Keywords:** biomarker, diabetic kidney disease, IL-33, inflammation, phase 2b, tozorakimab

## Abstract

**Introduction:**

Inflammation is a significant contributor to cardiorenal morbidity and mortality in diabetic kidney disease (DKD). The pathophysiological mechanisms linking systemic, subacute inflammation and local, kidney injury-initiated immune maladaptation is partially understood.

**Methods:**

Here, we explored the expression of proinflammatory cytokines in patients with DKD; investigated mouse models of type 1 and type 2 diabetes (T2D); evaluated glomerular signaling *in vitro*; performed *post hoc* analyses of systemic and urinary markers of inflammation; and initiated a phase 2b clinical study (FRONTIER-1; NCT04170543).

**Results:**

Transcriptomic profiling of kidney biopsies from patients with DKD revealed significant glomerular upregulation of interleukin-33 (IL-33). Inhibition of IL-33 signaling reduced glomerular damage and albuminuria in the uninephrectomized *db/db* mouse model (T2D/DKD). On a cellular level, inhibiting IL-33 improved glomerular endothelial health by decreasing cellular inflammation and reducing release of proinflammatory cytokines. Therefore, FRONTIER-1 was designed to test the safety and efficacy of the IL-33–targeted monoclonal antibody tozorakimab in patients with DKD. So far, 578 patients are enrolled in FRONTIER-1. The baseline inflammation status of participants (*N* > 146) was assessed in blood and urine. Comparison to independent reference cohorts (*N* > 200) validated the distribution of urinary tumor necrosis factor receptor 1 (TNFR1) and C-C motif chemokine ligand 2 (CCL2). Treatment with dapagliflozin for 6 weeks did not alter these biomarkers significantly.

**Conclusion:**

We show that blocking the IL-33 pathway may mitigate glomerular endothelial inflammation in DKD. The findings from the FRONTIER-1 study will provide valuable insights into the therapeutic potential of IL-33 inhibition in DKD.

## Graphical abstract

**Figure:**
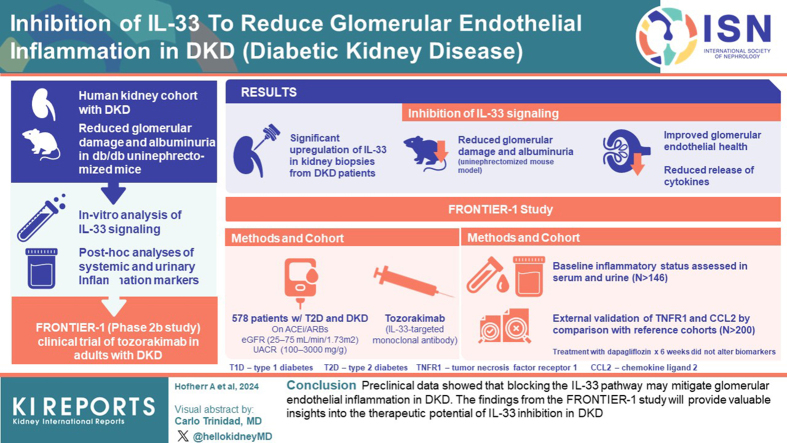

In DKD, various immune cell populations^1^ and several circulating cytokines have been correlated with poor cardiorenal outcomes and mortality, including neutrophils,^2^ eosinophils,^3^ CD4^+^ T cells,^4^ IL-1 α/β, IL-6, C-reactive protein (CRP), TNF α, TNFR1/2, and CCL2.^5^, ^6^, ^7^, ^8^, ^9^, ^10^, ^11^, ^12^, ^13^, ^14^ The distribution of risk associated with these biomarkers has indicated that inflammation plays a clinically relevant, disease-modifying role in at least 20% to 40% of patients with DKD.^1^^,^^3^, ^4^, ^5^^,^^7^, ^8^, ^9^, ^10^, ^11^, ^12^, ^13^, ^14^ For these patients, it is hypothesized that continuous inflammation impairs a broad range of biologic activities, from cell survival and proliferation to fibrosis and cell death. Although the associated pathophysiological mechanisms are still only partially understood, treating chronic subclinical inflammation may offer an important opportunity to attenuate residual cardiorenal disease progression in addition to current standard of care in patients with DKD regardless of albuminuria status.^7^^,^^15^

Valuable clinical data for the potential cardiovascular benefit of therapeutic targeting of specific inflammatory pathways in patients with chronic kidney disease (CKD) was derived from the Canakinumab Anti-Inflammatory Thrombosis Outcomes Study.^16^^,^^17^ This study tested a human IgGκ monoclonal antibody targeting IL-1β in adults with a history of myocardial infarction and systemic inflammation (*N* = 10,061; elevated high-sensitivity CRP > 2 mg/ml).^16^ *Post hoc* analysis of the 1875 participants with moderate CKD (30 ≤ estimated glomerular filtration rate [eGFR] < 60 ml/min per 1.73 m^2^) confirmed that this group had a higher absolute risk of major adverse cardiovascular events than participants with normal kidney function (6.92 vs. 4.13/100 person-years; *P* < 0.0001) and indicated that canakinumab particularly benefited patients with CKD (46% with diabetes) with the most robust antiinflammatory response to treatment (hazard ratio = 0.82 with *P* = 0.05 vs. hazard ratio = 0.68 with *P* = 0.0015 in patients achieving on-treatment high-sensitivity CRP levels < 2 mg/ml).^16^^,^^17^ In addition to IL-1β, other inflammatory targets have been explored in earlier-phase clinical studies in DKD, including IL-6, nuclear factor erythroid 2-related factor 2, apoptosis signal-regulating kinase 1, Janus kinase 1/2, and chemokine (C-C motif) receptors 2/5.^15^ However, despite some positive observations on urinary albumin excretion, it has been difficult to establish a clear association of these biomarkers with kidney function and unfavorable kidney outcomes.^15^ Therefore, an antiinflammatory medicine with a high efficacy that addresses the vicious cycle of systemic, subacute inflammation and local, kidney injury-initiated immune maladaptation has remained elusive.^18^

From a drug development standpoint, the prediction of kidney disease progression in patients with T2D also remains a substantial challenge.^19^ Clinical outcomes in DKD are extremely variable, with glycemic control, hypertension, albuminuria, and inflammation determining individual disease progression.^19^ Reliable identification of those patients with DKD, who are most likely to benefit from an antiinflammatory treatment will, therefore, be an important requirement for the successful development of targeted therapies.^15^

Here, we used candidate target profiling of several key inflammatory targets to identify IL-33 as an overexpressed inflammatory cytokine in human CKD. Furthermore, we: (i) show the significant benefit of inhibiting IL-33 signaling in a rodent model of DKD; (ii) demonstrate the mechanistic link between IL-33, eosinophils, and endothelial release of cardiorenal risk factors, such as TNFR1 and CCL2; (iii) investigate urine and systemic inflammatory biomarkers in patients with DKD; and, (iv) describe the ongoing phase 2b FRONTIER-1 clinical trial (NCT04170543), which is evaluating the clinical risk and benefit profile of the IL-33-targeted monoclonal antibody tozorakimab (also known as MEDI3506)^20^ for the treatment of DKD associated with inflammation.

## Methods

Details of the methods are described in the Supplementary Methods.

### Evaluation of Kidney Biopsies

#### Targeted Analysis of Inflammatory Gene Expression

Data from the Karolinska Institute/Sahlgrenska University Hospital human kidney cohort were used to analyze inflammatory marker expression in patients with DKD.^21^

#### Kidney Expression of IL-33 in Patients With DKD

Human kidney samples were processed according to the European Renal cDNA Bank protocol as previously described.^22^^,^^23^

### Immunohistochemistry

Fixed 3 to 4 μm human kidney sections were stained on an automated immunohistochemistry robot (Flatbed Autostainer Plus, Dako; Santa Clara, CA).

### mRNA-Based Assessment of IL-33 Pathway Signature

Gene-set variation analysis was performed on data from human renal biopsies from the European Renal cDNA Bank cohort and a signature score (scaled as –1 ≤ × ≤1) was calculated based on expression data from a predefined gene set (*IL-33*, *IL-1 receptor-like 1* [*ST2*], *IL-1 receptor accessory protein*, *TNFR*–*associated factor* 6, *myeloid differentiation primary response 88*, and *IL-1 receptor–associated kinase 4 [IRAK-4]*).^22^^,^^24^^,^^25^

### Mice

#### db/db Uninephrectomy

Male *db/db* mice (BKS.Cg-Dock7m +/+ Leprdb/J homozygotes; Charles River, Italy) were investigated in the Medimmune Biological Sciences Unit (Cambridge, UK). All animal protocols and procedures complied with the guidelines and regulations of a UK Home Office Project Licence, which had been reviewed and approved by the local Animal Welfare and Ethical Review Body.

#### Body Mass, Treatment Exposure, Creatinine, Albuminuria, and Hemoglobin A_1c_

Body mass was monitored once every 2 or 3 days and other parameters were measured at 10 (baseline), 13, and/or 15 weeks of age.

#### Histologic Analysis

Kidneys were collected at study termination. Thirty to 40 glomeruli were scored per kidney from the upper half of the section throughout the full thickness of the cortex. Mice were scored 0 to 4, based on extent of Periodic acid-Schiff (PAS)-positive mesangial expansion, scarcity of nuclei, and decrease in capillary luminal spaces.

#### Streptozotocin High-Fat Diet

This study was conducted by RenaSci Limited (Nottingham, UK) in accordance with the project license and Animals (Scientific Procedures) Act 1986.

#### IL-33 Detection

Mouse IL-33 protein levels from kidney homogenates were quantified using the MILLIPLEX Mouse TH17 Magnetic Bead Panel (MTH17MAG-47K, Merck Millipore; Burlington, MA).

Human IL-33 protein levels from cell lysates were measured using a U-PLEX Human IL-33 Assay (K151WFK, Meso Scale Discovery; Rockville, MD).

### *In Vitro* Analysis of IL-33 Signaling

#### Cell Culture

Human glomerular microvascular endothelial cells (HGMECs; ACBRI 128, Cell Systems; Kirkland, WA), human kidney mesangial cells (3031, Novabiosis; Durham, NC), and human renal proximal tubule epithelial cells (PTECs; CC-2553, Lonza; Basel, Switzerland) were grown to confluence and harvested with accutase (L11-007, PAA Laboratories; Pasching, Austria).

#### Recombinant IL-33 Protein Production

Cloning, expression, and purification of wild-type human IL-33 (amino acid [aa] 112–270) and a variant with all 4 cysteine residues mutated to serine (IL-33C>S) were synthesized as described previously.^26^

#### Mitogen-Activated Protein Kinase Assay

Phosphorylated mitogen-activated protein kinases, p38, and c-Jun N-terminal kinase were detected using a Meso Scale diagnostic assay (Phospho-p38 kit, K150CYD, Meso Scale Discovery; Phospho-JNK kit, K150CUD, Meso Scale Discovery, respectively).

#### Nuclear Factor κ-Light-Chain-Enhancer of Activated B Cells Translocation

Nuclear factor κ-light-chain-enhancer of activated B cells translocation to the nucleus was measured by immunofluorescence.^27^

#### Cytokine Detection

Proinflammatory cytokines were detected using a Meso Scale diagnostic assay (V-PLEX Proinflammatory Panel kit, K15049D or K15053D, Meso Scale Discovery; R-PLEX Human TNF-RI Assay, K1510VR, Meso Scale Discovery; and V-PLEX Human MCP-1 kit, K151NND, Meso Scale Discovery).

#### Proliferation Assays

Cells were treated with IL-33 or platelet-derived growth factor-BB (100-14B-2UG, PeproTech), or epidermal growth factor (236-EG-200, R&D Systems) used as positive controls. 5-ethynyl-2’-deoxyuridine incorporation was assessed using Amplex UltraRed reagent and the Click-iT 5-ethynyl-2’-deoxyuridine Proliferation Assay (C10499, Invitrogen).

#### Quantitative Polymerase Chain Reaction

Reverse transcription quantitative polymerase chain reaction, TaqMan RNA-to-CT 1-Step Kit (4392938, Thermo) was used together with an *IL-33* FAM probe (Hs04931857_m1, Thermo) or *ST2* (Hs00249384_m1, Thermo) and a *GAPDH* VIC probe (Hs99999905_m1, Thermo) or a murine *il-33* FAM probe (Mm00505403_m1, Thermo) and a murine *gapdh* VIC probe (Mm99999915_g1, Thermo). The ΔΔCT method was used to calculate the relative gene expression of samples.

### Reference Cohorts for Exploratory Biomarker Analysis

In Supplementary Table S1, we describe the reference cohorts used in the exploratory biomarker analysis.

### FRONTIER-1: Tozorakimab in Adults With DKD

The complete protocol information is provided in the Supplementary Material.

### Statistics

Data are presented as mean ± SD or as mean ± SEM. The Mann-Whitney *U* test was used to compare data. Correlation analysis was carried out using Spearman correlation. Where possible, a 1-way (Kruskal-Wallis) or a 2-way analysis of variance was carried out to compare experimental groups. Statistical significance was depicted as ∗*P* < 0.05, ∗∗*P* < 0.01, ∗∗∗*P* < 0.001, or ∗∗∗∗*P* < 0.0001.

## Results

### Profiling of Kidney Biopsies from Patients with DKD Indicated a Significant Glomerular Upregulation of IL-33 Signaling

**Figure 1:**
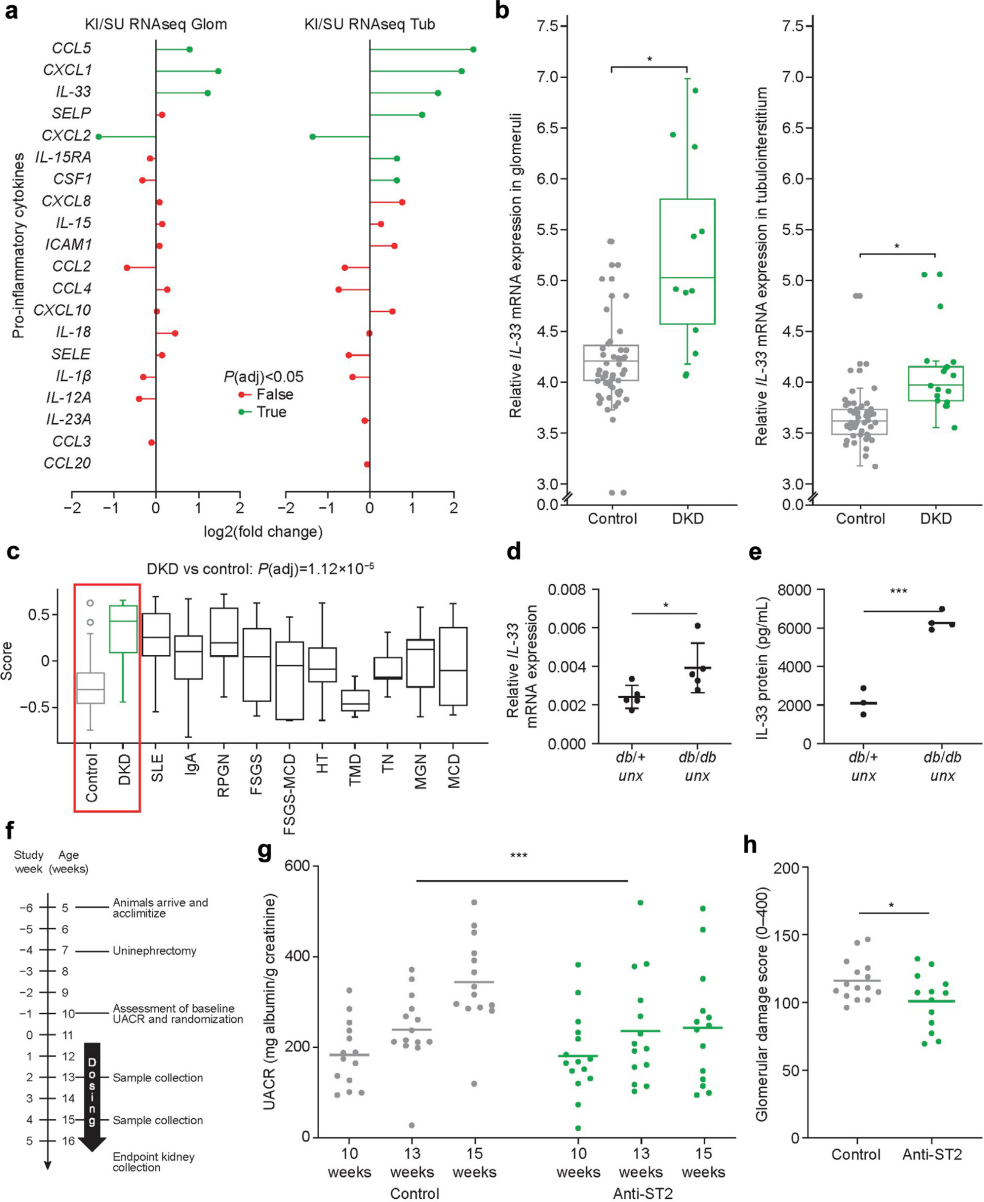
Expression of IL-33 in patients with DKD and inhibition of IL-33 signaling in DKD mouse models. (a) Differentially expressed cytokines relevant to CKD in ascending order of the lowest *P*-value in the glomerular and tubulointerstitial compartments with significantly differentially expressed cytokines colored green. (b) *IL-33* mRNA expression in patients with DKD relative to healthy control living donors in glomeruli (Wilcoxon, *P* = 7.4 × 10^–5^) and tubulointerstitium (Wilcoxon, *P* = 4.6 × 10^–6^) in the ERCB cohort. Boxes show the IQR; the middle horizontal line is the median and the whiskers indicate the minimum and maximum values. Individual data points are shown. (c) GSVA calculated signature score (–1 ≤ *x* ≤1) based on the redefined IL-33 signature gene set. The box plot shows the distribution of GSVA scores for each CKD condition. Boxes show the IQR; whiskers indicate the maximum, 1.5 × IQR. Outliers beyond 1.5 × IQR are plotted individually. Renal IL-33 (d) mRNA and (e) protein expression in diabetic uninephrectomized (*db*/*db unx*) and nondiabetic uninephrectomized (*db*/*+ unx*) mice at 16 weeks of age. (f) *In vivo* study design: mice underwent uninephrectomy at 7 weeks of age and were randomized by UACR at 10 weeks of age. Between 11 and 16 weeks of age, intraperitoneal doses of either isotype control or anti-ST2 antibodies (10 mg/kg) were administered 3 times per week. Urine samples were collected at 10, 13, and 15 weeks. Effect of anti-ST2 antibody on UACR and glomerular damage are shown in (g) and (h), respectively. The linear mixed-effect with trend model was used for the statistical assessment of albuminuria progression. Individual data points are shown, and horizontal lines represent the mean. ∗*P* < 0.05, ∗∗∗*P* < 0.001. DKD, diabetic kidney disease; ERCB, European Renal cDNA Bank; FSGS, focal segmental glomerulosclerosis; GSVA, gene-set variation analysis; HT, hypertensive nephropathy; IgA, immunoglobulin A nephropathy; IL-33, interleukin-33; IQR, interquartile range; MCD, minimal change disease; MGN, membranous glomerulonephritis; RPGN, rapidly progressive glomerulonephritis; SLE, systemic lupus erythematosus; TMD, thin basement membrane disease; TN, tumor nephrectomy; UACR, urinary albumin-to-creatinine ratio; *unx*, *uninephrectomized*.

Targeting specific signaling pathways rather than using broad antiinflammatory mechanisms is important to optimize efficacy and minimize side effects.^28^ Therefore, we explored the mRNA expression of 20 mechanistically diverse proinflammatory cytokines in kidney biopsies from patients with DKD.^15^ *Post hoc* analysis of the Karolinska Institute/Sahlgrenska University Hospital DKD patient cohort receiving angiotensin-converting enzyme inhibitor or angiotensin II receptor blockers (*N* = 19) demonstrated a significant upregulation of *CCL5,* *chemokine* (*C-X-C motif*) *ligand 1* (*CXCL1*), and *IL-33* in glomerular and tubulointerstitial compartments (Figure 1a).^21^^,^^24^^,^^29^

IL-33 is a potent mediator of injury-induced local inflammation that has been shown to activate expression of *CCL5* and *CXCL1* (C-X-C motif chemokine receptor 2 [CXCR2] ligand as IL-8) significantly.^30^, ^31^, ^32^, ^33^, ^34^ Consequently, we further investigated the upregulation of *IL-33* in 2 additional diverse patient cohorts.^35^ These samples (*n* = 48) confirmed a significant upregulation of *IL-33* mRNA in the kidneys from patients with DKD on standard-of-care compared with kidneys from healthy organ donors (Figure 1b and Supplementary Figure S1). Notably, this upregulation of *IL-33* correlated negatively with eGFR (Supplementary Figure S1). Initial histologic analysis also revealed elevated levels of IL-33 protein in a biopsy obtained from a patient with DKD (Supplementary Figure S2).

To assess the functional relevance of *IL-33* mRNA upregulation, we quantified IL-33 signaling by gene-set variation analysis.^25^ ST2, IL-1 receptor accessory protein, TNFR-associated factor 6, myeloid differentiation primary response 88, and IL-1 receptor–associated kinase 4 are known targets of IL-33.^36^^,^^37^ Expression analysis of this IL-33 signature indicated consistent pathway activation in the glomerular compartment, but no apparent activation in the tubulointerstitium (Figure 1c, Supplementary Figure S3, and Supplementary Table S2).

Significant glomerular activation of the IL-33 signature was also confirmed beyond DKD across most CKD etiologies, further implicating maladaptive IL-33 signaling as an important factor in kidney disease progression (Figure 1c).^38^, ^39^, ^40^, ^41^

### Inhibition of IL-33 Signaling Reduced Glomerular Damage and Albuminuria in the Uninephrectomized *db/db* Mouse Model of DKD

To investigate the pathophysiological relevance of IL-33 *in vivo*, we examined its activity in 2 distinct animal models that mechanistically represent type 1 diabetes and T2D, respectively: *high-fat diet Streptozotocin* and *db/db uninephrectomized (unx)*.^42^, ^43^, ^44^ Consistent with the elevated mRNA expression of *IL-33* in human disease, we found a significant increase in both IL-33 mRNA and protein expression in kidneys of mice with T2D with reduced kidney function (*db/db unx*; Figure 1d and e). Interestingly, Streptozotocin-induced type 1 diabetes mice did not exhibit a similar upregulation of IL-33 at 16 weeks, suggesting a distinct disease time course or divergence in inflammatory responses between type 1 diabetes and T2D in these models (Supplementary Figure S4).

Antibody-mediated inhibition of IL-33 signaling by neutralization of the IL-33 receptor, ST2, furthermore provided significant treatment benefit in animals with T2D.^45^^,^^46^ For this analysis, 7-week-old *db/db* mice were uninephrectomized and then randomized at week 10 by urinary albumin-to-creatinine ratio (UACR). Intraperitoneal treatment was administered at a dose of 10 mg/kg, 3 times per week, for 5 weeks with either mouse anti-ST2 (*n* = 13) or isotype control antibodies (*n* = 14) (Figure 1f).^45^ Treatment-consistent antibody exposure was confirmed at weeks 2 and 4 (Supplementary Figure S5). At the end of the study, kidneys were collected, and assessment of glomerular pathology performed by scoring of mesangial expansion, scarcity of nuclei, and decrease in capillary luminal spaces. In this progressive model of DKD, inhibition of IL-33/ST2 signaling translated into a significant protection against functional and structural kidney damage compared with control animals: progression of albuminuria reduced by 68.7% (*P* = 0.0001) and glomerular damage decreased by 13% (*P* = 0.0331) (Figure 1g and h). This prevention of renal disease progression was, furthermore, associated with a significantly reduced eosinophil percentage of leukocytes (−52%, *P* = 0.0293), a known consequence of IL-33 blockage (Supplementary Figure S6).^47^, ^48^, ^49^ Body weight, blood glucose, hemoglobin A_1c_, and urine volume were not affected by anti-ST2 treatment (Supplementary Figure S7).

### Mechanistically, Inhibiting IL-33 Improved Glomerular Endothelial Health by Decreasing Cellular Inflammation and Reducing Release of Proinflammatory Cytokines

**Figure 2:**
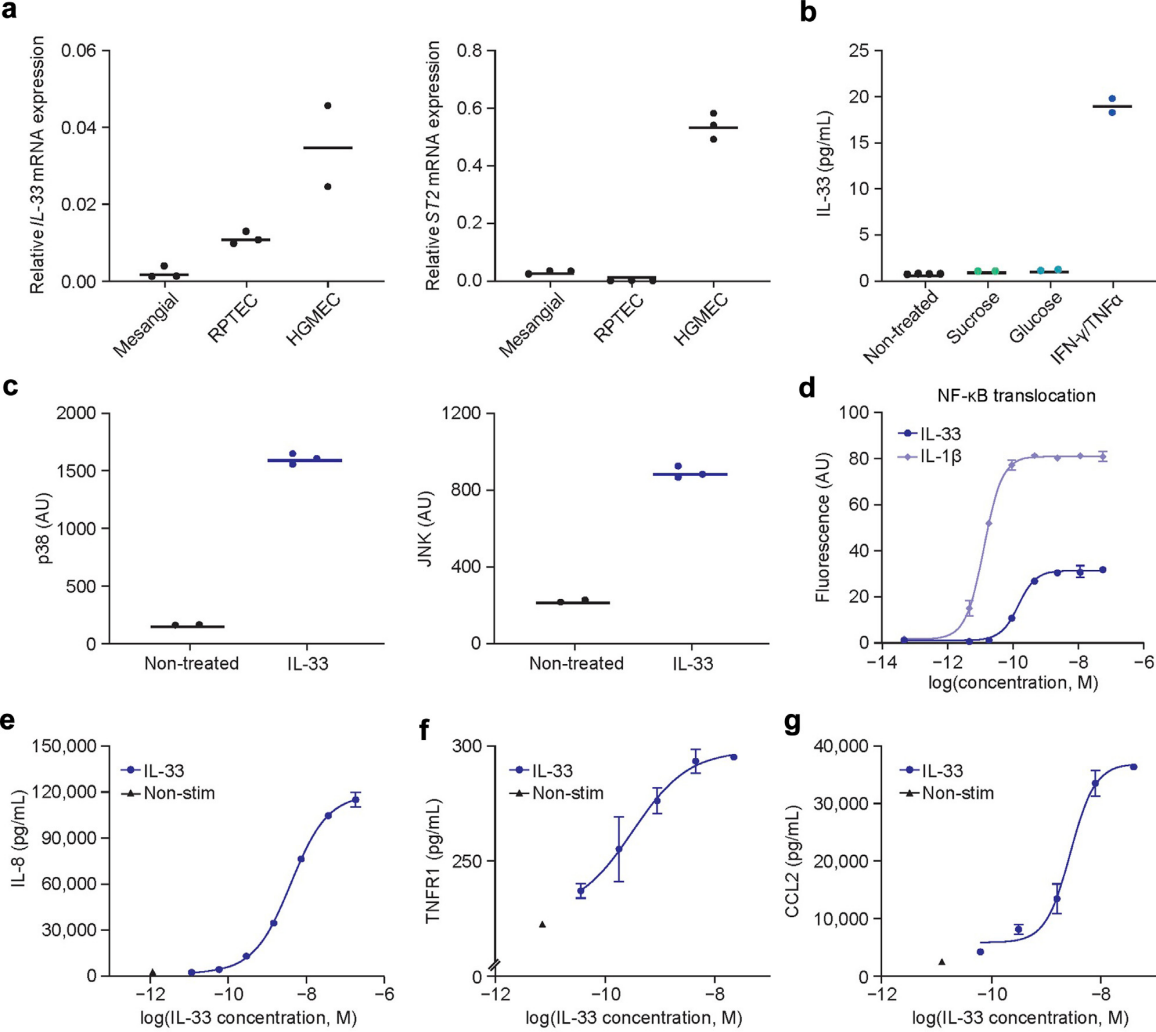
IL-33 signaling and downstream effects on HGMEC. (a) *IL-33* and *ST2* basal mRNA expression in primary human renal cells. (b) IL-33 intracellular protein expression in HGMEC in response to several exogenous stimuli. (c) and (d) Activation of inflammatory signaling pathways; MAP kinases p38 and JNK, and NF-κB nuclear translocation in HGMEC after exogenous IL-33 stimulation. Dose-response curves of proinflammatory cytokine secretion (e) IL-8, (f) TNFR1, and (g) CCL2, in HGMEC in response to IL-33. For (a) to (c), individual data points are shown, and horizontal lines represent the mean. For (d) to (g), error bars represent the SD. CCL2, C-C motif chemokine ligand 2; HGMEC, human glomerular microvascular endothelial cells; IFN-γ, interferon-γ; IL-8, interleukin-8; IL-33, interleukin-33; JNK, c-Jun N-terminal kinase; MAP, mitogen-activated protein; NF-κB, nuclear factor κ-light-chain-enhancer of activated B cells; non stim, nonstimulated; RPTEC, renal proximal tubule epithelial cells; TNFα, tumor necrosis factor α; TNFR1, tumor necrosis factor receptor 1.

**Figure 3:**
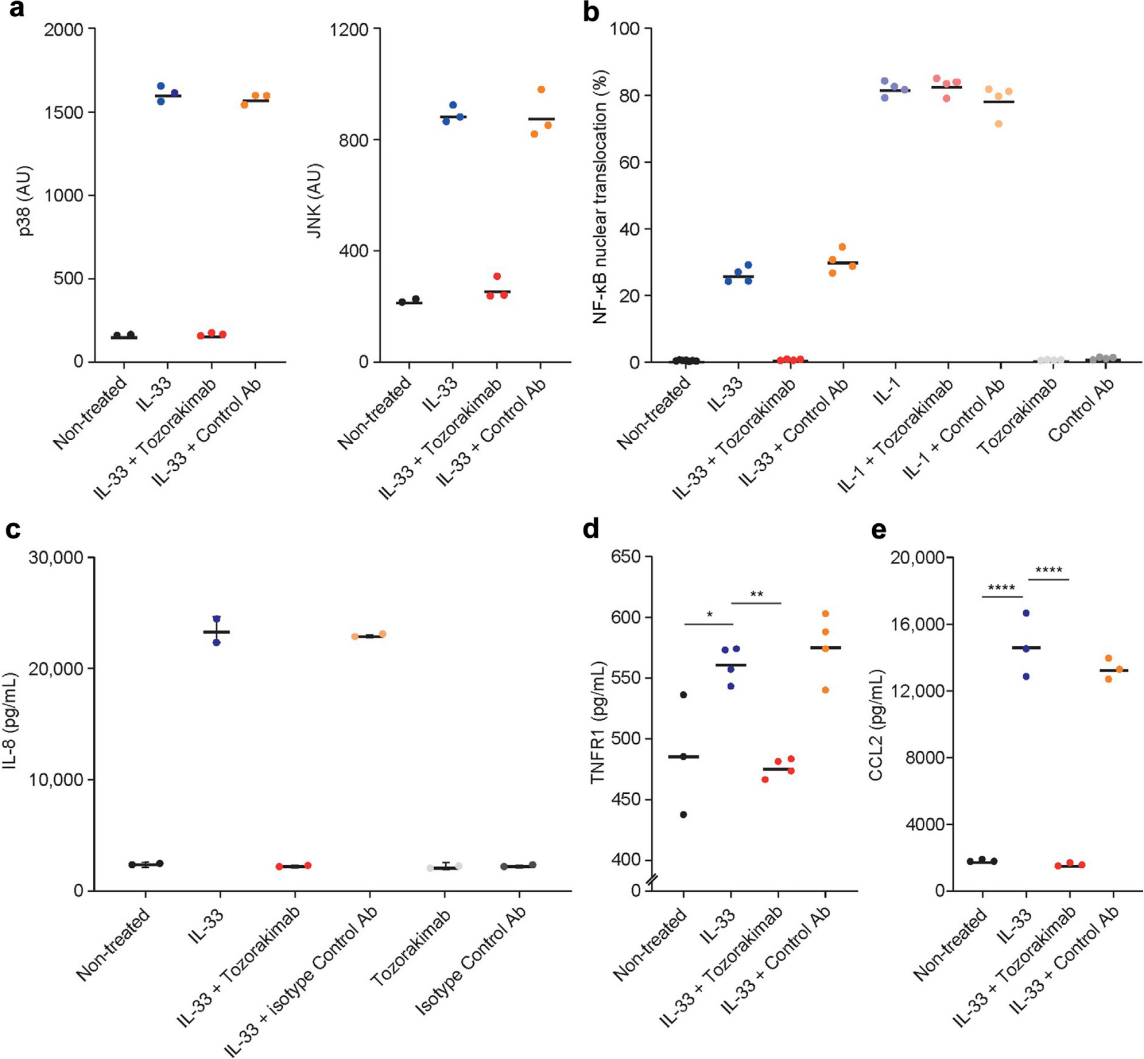
Tozorakimab inhibition of IL-33–induced proinflammatory response in HGMEC. Effect of tozorakimab or isotype control antibody treatment on the activation of inflammatory signaling pathways; MAP kinases (a) p38 and JNK, and (b) NF-κB nuclear translocation in HGMEC after exogenous IL-33 stimulation. Effect of tozorakimab or isotype control antibody treatment on the secretion of proinflammatory cytokines; (c) IL-8, (d) TNFR1, and (e) CCL2 in HGMEC after exogenous IL-33 stimulation. Individual data points are shown, and horizontal lines represent the mean. For (c), error bars represent the SD. ∗*P* < 0.05, ∗∗*P* < 0.01, or ∗∗∗∗*P* < 0.0001. CCL2, C-C motif chemokine ligand 2; HGMEC, human glomerular microvascular endothelial cells; IL-8, interleukin-8; IL-33, interleukin-33; JNK, c-Jun N-terminal kinase; MAP, mitogen-activated protein; NF-κB, nuclear factor κ-light-chain-enhancer of activated B cells; TNFR1, tumor necrosis factor receptor 1.

To clarify the mechanism of glomerular benefit involved in IL-33/ST2 inhibition, we next investigated the glomerular cell type mediating IL-33 signaling. In human glomeruli, *IL-33* mRNA expression is highest in endothelial cells (Supplementary Figure S8).^50^^,^^51^ Similarly, *in vitro*, we observed the most prominent IL-33/ST2 mRNA and IL-33 protein expression in HGMECs—expression in these cells was higher than in human mesangial cells and primary human renal proximal tubule epithelial cells (Figure 2a and Supplementary Figure S9). We therefore hypothesized that maladaptive IL-33/ST2 signaling may contribute to glomerular damage in T2D by causing endothelial dysfunction. This dysfunction has been previously functionally linked to podocyte injury, proteinuria, and mesangial matrix expansion.^52^ Supporting this hypothesis, we discovered an IL-33-mediated positive feedback loop between circulating factors of inflammation and endothelial dysfunction in HGMECs. Exogenous application of systemic factors of inflammation such as interferon-ɣ and TNFα was associated with a significant increase in IL-33 (Figure 2b). In addition, IL-33 was sufficient to activate pivotal intracellular pathways of inflammation, including mitogen-activated protein kinases (p38 and c-Jun N-terminal kinase) and nuclear factor κ-light-chain-enhancer of activated B cells (Figure 2c and d and Supplementary Figure S10A), as well as extracellular factors and proinflammatory cytokines such as IL-1β, IL-6, IL-8, TNFR1, and CCL2 (Figure 2e–g and Supplementary Figure S10B). Mesangial and epithelial cells, in contrast, did not show a robust inflammatory response to IL-33 (Supplementary Figure S11). Consistent with an IL-33–mediated glomerular endothelial immune-pathology in DKD, we observed that both the intracellular and extracellular effects of IL-33 were normalized after treatment with tozorakimab, a high-affinity IL-33–neutralizing immunoglobulin G1 monoclonal antibody developed by AstraZeneca (Figure 3a–e and Supplementary Figure S12).^20^

### Inhibition of IL-33 May Be Most Beneficial in Patients With DKD and Elevated Inflammatory Biomarkers

Taken together, the significant glomerular upregulation of IL-33 in patients with DKD, the substantial kidney-protective effect of suppression of IL-33/ST2 signaling in the *db/db unx* mouse model of DKD, and the molecular function of IL-33 as a significant glomerular amplifier of inflammation underscored the potential clinical benefit of IL-33 blockade by tozorakimab in patients with DKD. The previous association of IL-33 signaling-associated inflammatory biomarker with adverse cardiorenal outcomes furthermore helped to specify a precision medicine hypothesis: the clinical benefit of tozorakimab may be most pronounced in those patients with DKD who show the highest inflammatory activity.^5^^,^^7^, ^8^, ^9^, ^10^, ^11^, ^12^, ^13^, ^14^

However, optimal assessment of IL-33–mediated inflammatory activity and, so the disease-modifying potential of anti–IL-33 intervention, is complex in the multimorbid DKD patient population with competing risk factors.^19^ To start addressing this challenge, we next evaluated the potential of the following tozorakimab-responsive or outcome-relevant biomarkers for future patient selection (Figure 3, Supplementary Figure S6, and Supplementary Figure S12): IL-33, IL-1β, IL-6, CRP, IL-8, TNFR1, CCL2, and eosinophils.

It has been shown that direct assessment of free or active IL-33 in circulation is not feasible.^53^ All IL-33 in circulation is bound to the soluble form of its receptor, soluble ST2.^54^ Therefore, we developed an assay to measure the concentration of the IL-33 or soluble ST2 complex in serum.^20^ Ample data and standardized assays are available for IL-1β, IL-6, CRP, IL-8, and eosinophils. For TNFR1 and CCL2, we validated assays to explore serum and urine concentrations in patients with DKD (Supplementary Figure S13).

**Figure 4:**
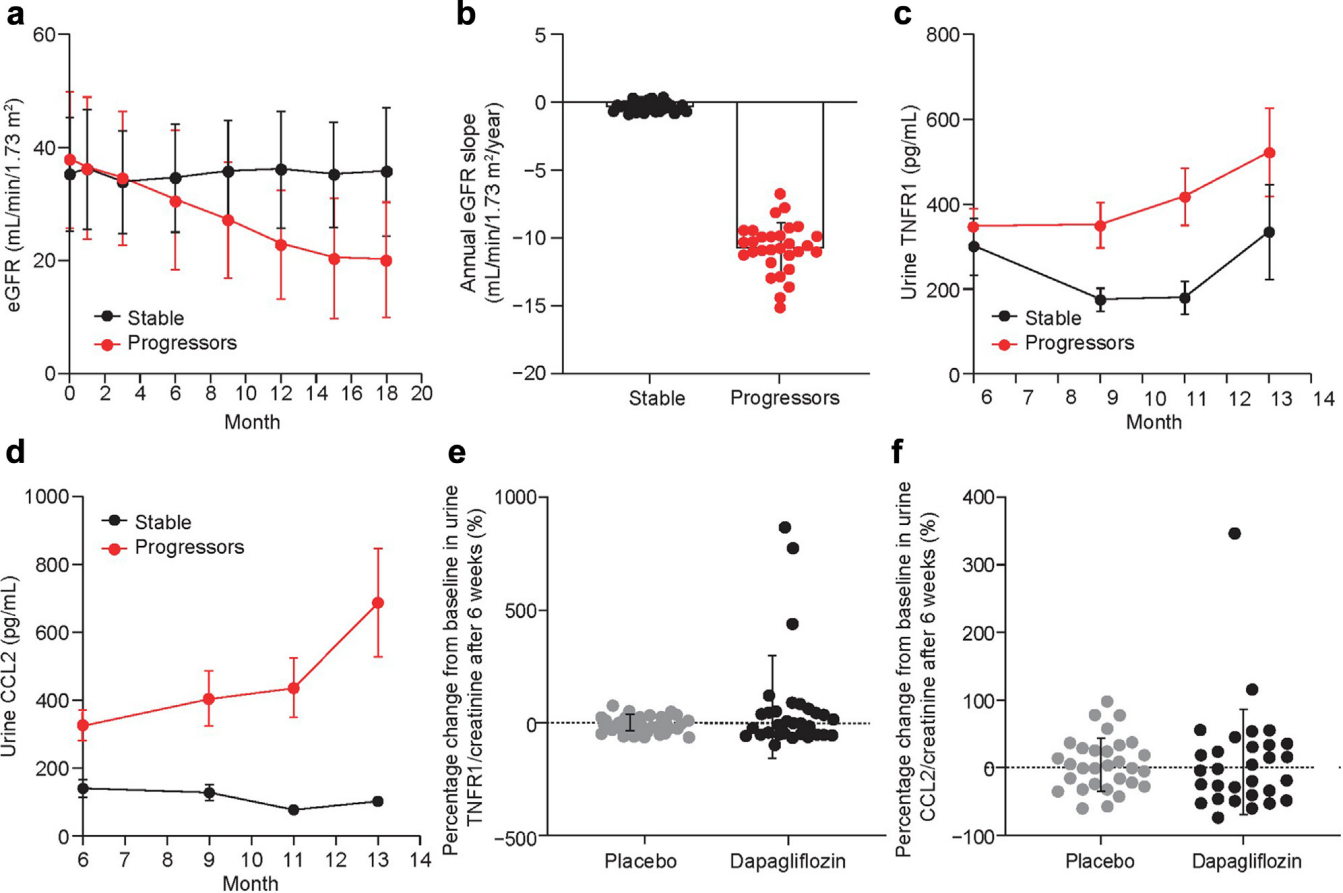
Assessment of urine inflammatory biomarkers in patients with DKD. (a) eGFR, (b) annual eGFR, (c) urine TNFR1, and (d) urine CCL2 of patients with DKD with either stable (black) or progressive loss of eGFR (red) from the Sun-MACRO study.^55^ Error bars represent the SD. Percentage change from baseline in urine (e) TNFR1/creatinine and (f) CCL2/creatinine in patients with DKD treated with dapagliflozin or placebo from the IMPROVE study.^56^ Individual data points are shown, and error bars represent SD. CCL2, C-C motif chemokine ligand 2; DKD, diabetic kidney disease; eGFR, estimated glomerular filtration rate; TNFR1, tumor necrosis factor receptor 1.

The potential clinical usefulness of the TNFR1 and CCL2 tests was furthermore investigated by retrospective comparison of patients from the Sun-MACRO study who had DKD and either stable kidney function over 18 months (*n* = 30; baseline eGFR = 35.2) or experienced progressive loss of eGFR > 5 ml/min/yr (*n* = 30; baseline eGFR = 37.9) (Figure 4a and b and Supplementary Figure S14).^55^ Interestingly, we observed that TNFR1 and CCL2 in urine were more effective in differentiating progressors from nonprogressors than respective serum concentrations (Figure 4c and d and Supplementary Figure S14).

Finally, because all human epidemiologic data used for this study were obtained from patients with DKD who were receiving only an angiotensin-converting enzyme inhibitor and/or angiotensin II receptor blockers as part of their treatment regimen, we investigated whether the recent inclusion of sodium-glucose cotransporter 2 inhibitors in the standard care for DKD may affect the potential effectiveness of tozorakimab treatment. To do this, we measured urinary TNFR1 and CCL2 *post hoc* in the IMPROVE clinical crossover study of the sodium-glucose cotransporter 2 inhibitor dapagliflozin in DKD (*n* = 33).^56^ We found that 6-week treatment of dapagliflozin did not alter the concentration of TNFR1 and CCL2 in urine significantly (Figure 4e and f).

### FRONTIER-1: A Phase 2b Study of Tozorakimab for the Treatment of Patients With DKD

**Figure 5:**
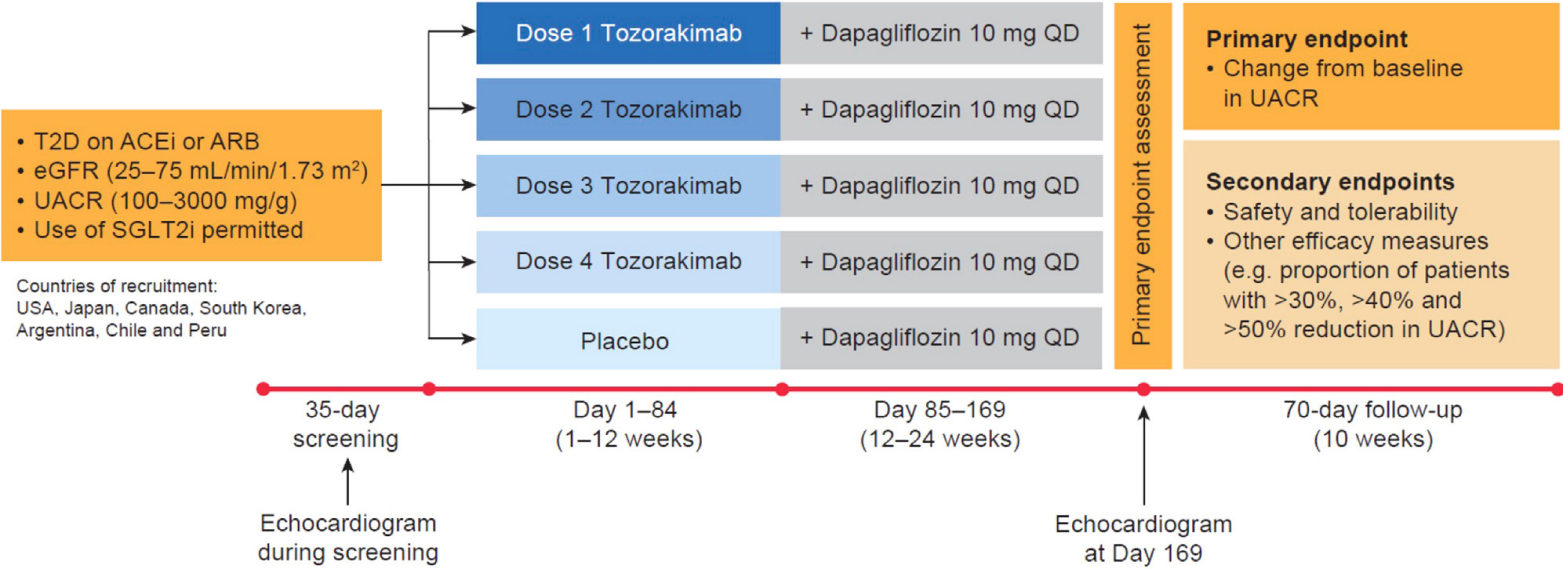
FRONTIER-1 phase 2b study design. Eligible patients were randomized to receive tozorakimab or placebo once every 4 weeks for 24 weeks. All participants received dapagliflozin 10 mg once daily from 12 to 24 weeks. Participants were followed-up with for 10 weeks. ACEi, angiotensin-converting enzyme inhibitor; ARB, angiotensin receptor blocker; eGFR, estimated glomerular filtration rate; Q4W, every 4 weeks; QD, once daily; SGLT2i, sodium-glucose cotransporter 2; T2D, type 2 diabetes; UACR, urinary albumin-to-creatinine ratio.

Recently, IL-33 inhibition has been successfully studied in early clinical trials for patients with moderate-to-severe asthma and in former smokers with chronic obstructive pulmonary disease.^47^, ^48^, ^49^ Immunomodulation by anti–IL-33 antibody intervention was well-tolerated and had a high efficacy in preventing disease exacerbation in both patient populations.^47^, ^48^, ^49^ Owing to the encouraging preclinical and epidemiologic data on IL-33 in patients with DKD, a phase 2b clinical study was designed to test the safety and efficacy of tozorakimab in these patients (Figure 5). The key considerations for the FRONTIER-1 study design were as follows:

1. Primary evaluation of albuminuria as a surrogate for progression of DKD (Figure 1g and h).
2. A reduction of >21% to 27% in UACR over a 6-month follow-up period in participants with UACR ≥30 mg/g has been shown to give a reasonable prediction of a nonzero benefit on adverse clinical kidney outcomes.^57^
3. Individual albuminuria is highly variable (here, we assume an SD in log-transformed UACR of 1).^58^
4. Current standard-of-care, including angiotensin-converting enzyme inhibitor, angiotensin II receptor blockers, and sodium-glucose cotransporter 2 inhibitor, does not seem to reduce the inflammation-associated risk in DKD (Figure 4e and f).
5. Noninterventional studies of inflammatory biomarkers in DKD suggest that 20% to 40% of patients are expected to benefit significantly from treatment with tozorakimab.^5^^,^^7^, ^8^, ^9^, ^10^, ^11^, ^12^, ^13^, ^14^
6. Detailed responder analysis based on inflammatory biomarker in blood and urine.

### FRONTIER-1: Patient Demographics and Clinical Characteristics at Baseline

Between November 2019 and March 2023, 1510 patients were screened, and 578 patients were randomized and treated. Baseline characteristics were in line with our expectations for this patient population (Table 1). The mean (± SD) age was 66.6 ± 9.8 years, and 172 participants (29.8%) were female. At baseline, most patients (98.3%) were receiving angiotensin-converting enzyme inhibitor or angiotensin II receptor blockers, and 26.5% were receiving sodium-glucose cotransporter 2 inhibitor. The mean eGFR was 47.8 ml/min per 1.73 m^2^ (± 14.7) and the median UACR was 465.4 mg/g (interquartile range = 7–5709) (Table 1).

**Table 1:** FRONTIER-1 patient demographics and baseline characteristics (full analysis population)

Characteristic	Participants (*N* = 578)
Age (yr), mean ± SD	66.6 ± 9.8
Female, *n* (%)	172 (29.8)
Race, *n* (%)	578 (100)
White	366 (63.3)
Black	50 (8.7)
Asian	130 (22.5)
Other	11 (1.9)
Body mass index, mean ± SD	31.6 ± 6.6
Systolic pressure (mm Hg), mean ± SD	134.4 ± 11.9
Diastolic pressure (mm Hg), mean ± SD	75.7 ± 9.7
Duration of diabetes (yr), mean ± SD	17.5 ± 9.7
HbA_1c_ (%), mean ± SD	7.56 ± 1.15
eGFR (ml/min per 1.73 m^2^), mean ± SD	47.8 ± 14.7
CKD, *n* (%)	578 (100)
UACR (mg/g), mean ± SD	764.6 ± 773.7
UACR (mg/g), geometric mean (CV%)	457.9 (101.2)
UACR group, *n* (%)	
<30	5 (0.9)
30–300	211 (36.5)
>300	361 (62.5)
CRP, mean ± SD	0.385 ± 0.702
Hematology, mean ± SD	
Basophils (×10^3^/ul)	0.058 ± 0.033
Eosinophils (×10^3^/ul)	0.216 ± 0.170
Hematocrit (%)	39.3 ± 5.3
Hemoglobin (g/dl)	12.94 ± 1.75
Erythrocytes (×10^6^/ul)	4.39 ± 0.60
Leukocytes (×10^3^/ul)	7.142 ± 1.860
Lymphocytes (×10^3^/ul)	1.770 ± 0.574
Monocytes (×10^3^/ul)	0.394 ± 0.148
Neutrophils (×10^3^/ul)	4.703 ± 1.524
Platelets (×10^3^/ul)	241.7 ± 66.3
Medication, *n* (%)	
Insulin (fast-acting)	25 (4.3)
Insulin (intermediate-acting and long-acting)	166 (28.7)
ACEi or ARB	568 (98.3)
SGLT2i	153 (26.5)

ACEi, angiotensin-converting enzyme inhibitor; ARB, angiotensin II receptor blocker; CKD, chronic kidney disease; CRP, C-reactive protein; CV, coefficient of variation; eGFR, estimated glomerular filtration rate; HbA_1C_, hemoglobin A1C; SGLT2i, sodium-glucose cotransporter 2 inhibitor; UACR, urinary albumin-to-creatinine ratio.

Data cutoff: March 22, 2023.

**Figure 6:**
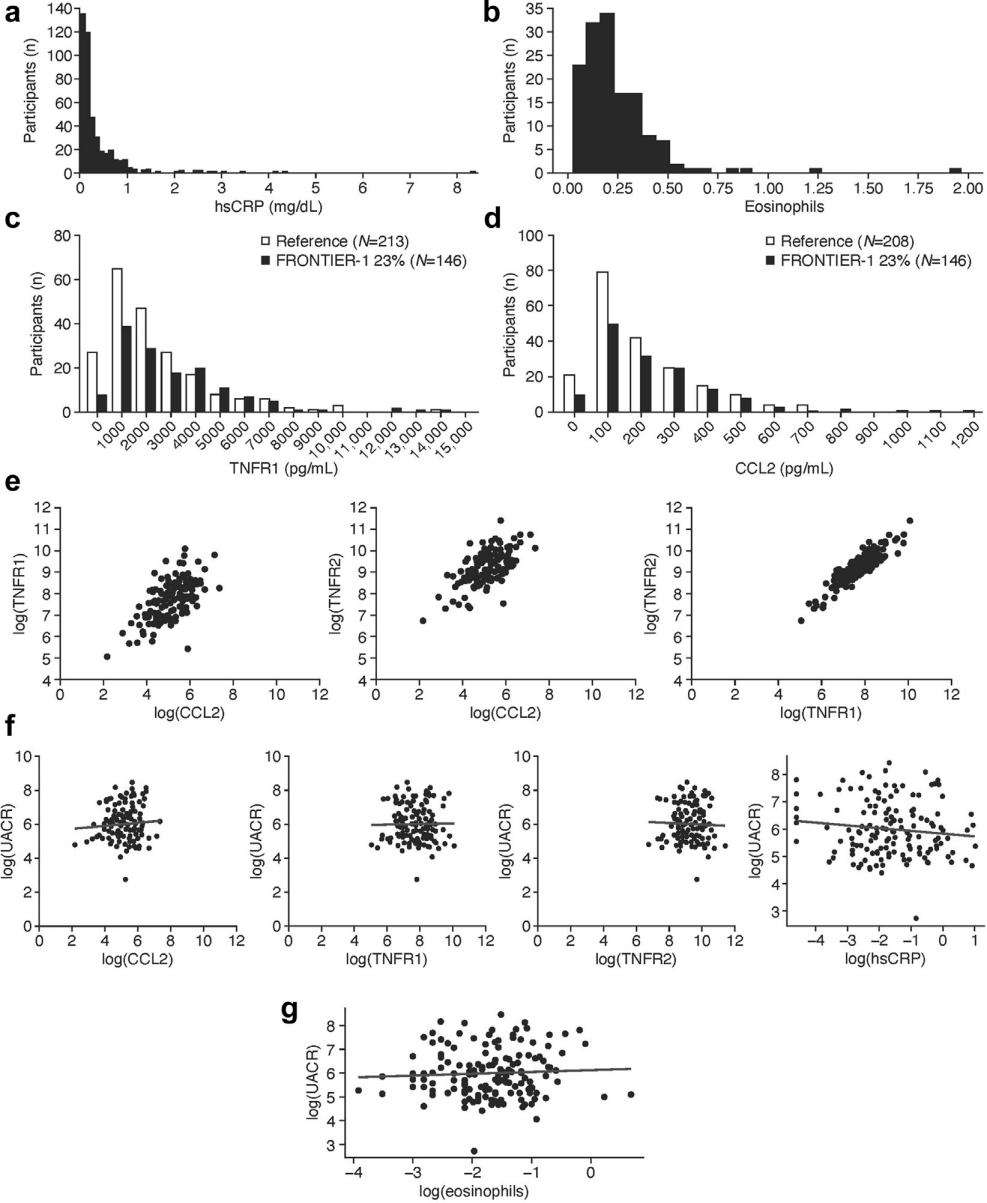
Inflammation profiles for participants in FRONTIER-1. Baseline expression of (a) hsCRP, (b) eosinophils, (c) TNFR1, and (d) CCL2 across participants in the FRONTIER-1 trial (black bars). (b) and (c) also show expression distributions in participants from independent cohorts for comparison (white bars). (e) Correlations between log-transformed CCL2, TNFR1, and TNFR2. (f) Correlations between log-transformed UACR and log-transformed CCL2 (Pearson’s R, 0.106), TNFR1 (−0.00859), TFR2 (−0.0257) or hsCRP (−0.118). (g) Correlation between log-transformed baseline UACR and eosinophils (Pearson’s R, 0.0555). CCL2, C-C motif chemokine ligand 2; hsCRP, high-sensitivity C-reactive protein; MCP-1, monocyte chemoattractant protein-1; TNFR1, tumor necrosis factor receptor 1; TNFR2, tumor necrosis factor receptor 2; UACR, urinary albumin-to-creatinine ratio.

In a first exploratory analysis, the baseline inflammatory status of participants was evaluated in circulation and in urine by assessment of eosinophils, CRP, TNFR1, and CCL2 (*n* > 146) (Figure 6a–d and Supplementary Figures S15–S20). To provide an external validation of the distributions of TNFR1 and CCL2, we additionally tested samples from 213 patients with DKD from 4 independent cohorts (Figure 6c and d and Supplementary Table S1).^55^^,^^56^^,^^59^ These data provide a first comparison of urinary TNFR1 and CCL2 across cohorts and confirm uniform distributions of respective biomarker levels (Figure 6c and d). Furthermore, our study demonstrated a significant correlation between the urinary biomarkers of inflammation (Figure 6e). Yet, the levels of eosinophils, CRP, TNFR1/2, and CCL2 showed only a limited association with albuminuria in all-comers (Figure 6f and g and Supplementary Figures S21–S23). Similar to what has been found in previous studies, this observation suggests the existence of an additional, independent cardiorenal risk component associated with these markers.^5^, ^6^, ^7^, ^8^, ^9^, ^10^, ^11^, ^12^, ^13^, ^14^

Importantly, however, the observed similarity in urine levels suggested consistent recruitment of inflamed and noninflamed patients in DKD clinical cohorts. Therefore, by conducting a retrospective responder analysis in FRONTIER-1, we hope to identify those high-risk patients with DKD who are most likely to benefit from immunomodulatory treatment with tozorakimab.

## Discussion

In DKD, chronic activation of the immune system is associated with progression of kidney failure and major adverse cardiovascular events independent of albuminuria.^18^^,^^60^^,^^61^ It is hypothesized that reducing the severity of this pathology could yield significant clinical benefits for affected individuals.^7^^,^^15^ However, despite substantial mechanistic insights, an efficacious, well-tolerated treatment of inflammation-dependent cardiorenal disease progression has remained elusive.^7^^,^^15^ Here, we show that inhibition of IL-33 may provide an opportunity to address this unmet medical need on top of standard of care.

IL-33 was discovered as an endothelial cell nuclear factor that is mostly expressed in large and small blood vessels in almost all human organs at steady state.^62^, ^63^, ^64^ Later, significant expression was also confirmed in epithelial cells from barrier tissues and fibroblastic stromal cells;^65^ and association studies have established a genome-wide significant association between variants in IL-33, eosinophil numbers, and risk for asthma or chronic obstructive pulmonary disease.^47^^,^^66^^,^^67^

The main function of IL-33 is to signal the immune system about the presence of acute damage and initiate a response to repair and remove damaged tissue.^31^ Structurally, IL-33 is a 270 aa, IL-1 family cytokine with 3 distinct protein domains as follows: (i) a chromatin-binding domain to store the inactive IL-33 cytokine in the nucleus (aa 1–65); (ii) a protease sensor domain (aa 66–111), which is important for cleavage-mediated extracellular activation after tissue injury; and (iii) an IL-1-like cytokine effector domain with autocrine and paracrine activity (aa 112–270).^68^, ^69^, ^70^, ^71^ In contrast to the healthy alarmin function in tissue homeostasis, chronic release of IL-33 has been associated with immune maladaptation and fibrosis in various organs, including the lung, liver, heart, kidney, and skin.^31^

Studies in animal models have shown that prolonged release of IL-33 contributes to disease progression or exacerbation by pleiotropic overactivation of immune cells, including T helper type 2 cells and innate lymphoid type 2 cells, and subsequent production of profibrotic cytokines.^31^ Accumulation of extracellular matrix proteins is, furthermore, amplified by a direct, IL-33–mediated, activation of fibroblasts and myofibroblasts, the main cell types responsible for extracellular matrix production and remodeling.^31^ Encouragingly, the potential clinical benefit of IL-33 blockade has been indicated by phase 2 clinical studies in patients with moderate-to-severe asthma and in former smokers with chronic obstructive pulmonary disease.^47^^,^^49^^,^^72^ In those patients, inhibition of IL-33 in addition to standard-of-care reduced eosinophils by up to 40%, improved lung function, and reduced exacerbation rate significantly.^47^^,^^49^^,^^73^ Anti–IL-33 treatment was generally well-tolerated, with an acceptable safety profile, and incidences of treatment-emergent adverse events, serious adverse events, and death similar to placebo.^47^^,^^49^

In the context of the kidney, IL-33 has been implicated in the development and progression of several diseases etiologies, including acute kidney injury, CKD, glomerulonephritis, and renal allograft fibrosis.^38^^,^^40^^,^^50^^,^^74^, ^75^, ^76^, ^77^, ^78^ In several mouse models of renal disease, blocking IL-33 has also been shown to reduce disease severity.^74^^,^^75^^,^^79^, ^80^, ^81^ Our investigation of IL-33 in DKD has provided additional insight into the important immunomodulatory effect of this alarmin. We confirmed an IL-33–dependent paracrine activation of eosinophils in circulation and uncovered a significant autocrine signaling effect specifically on human glomerular microvascular endothelial cells, resulting in glomerular inflammation.^82^ Specifically, IL-33 was found to activate the critical mitogen-activated protein kinase or nuclear factor κ-light-chain–enhancer of activated B cells pathway and to stimulate the secretion of proinflammatory cytokines, including IL-8, TNFR1, and CCL2. Antibody-mediated systemic inhibition of IL-33 signaling in T2D mice with reduced kidney function and proteinuria (*db/db unx*) was then sufficient to reduce endothelial inflammation, improve glomerular health, and attenuate overall kidney disease progression.

Future research is needed to gain a full understanding of the complex molecular mechanisms underlying the role of IL-33 in the diabetic and nondiabetic kidney and cardiovascular disease. Particularly interesting is the recent characterization of an IL-33 oxidized form showing ST2-independent signaling through the receptor for advanced glycation end products or epidermal growth factor receptor complex.^20^^,^^26^^,^^83^ The pleiotropic proinflammatory function of IL-33 raises intriguing possibilities for the clinical translation of the observed benefits.

With tozorakimab in FRONTIER-1, we hope to protect glomerular health in patients with DKD by decreasing cellular inflammation. Studying the effects of tozorakimab in all-comers, as well as a predefined subgroup of patients with DKD and heightened inflammatory status, will yield a deeper understanding of the potential clinical advantages of this innovative compound. Tozorakimab is a human therapeutic monoclonal antibody that specifically binds to IL-33 with very high affinity, fully preventing IL-33 signaling of all redox forms by inhibition of IL-33 receptor binding (ST2 and receptor for advanced glycation end products or epidermal growth factor receptor complex).^20^ In phase 1 clinical testing, tozorakimab was generally well-tolerated, without discernable off-target signals.^72^ In addition to FRONTIER-1, tozorakimab is also being evaluated in preclinical and clinical studies for the treatment of other conditions such as symptomatic chronic obstructive pulmonary disease, moderate-to-severe asthma, acute respiratory failure, and atopic dermatitis.^84^, ^85^, ^86^

Taken together, our studies have confirmed inflammation as an important pathophysiological factor in DKD.^18^ We found a connection between IL-33 and the residual risk of kidney disease progression on top of standard-of-care in patients with DKD and significant subclinical inflammation. Limitations of our study include the observational and/or preclinical nature of the evidence suggesting that IL-33 plays a significant role in the regulation of kidney homeostasis. The FRONTIER-1 phase 2b clinical study is ongoing to evaluate the therapeutic potential of targeting IL-33 as biomarker-guided precision medicine in DKD.

## Disclosure

The study was designed, interpreted, and reported as a collaboration between the investigators and employees of AstraZeneca. AH, ELM, BM, AS, TS, JC, DB, BC, PBLH, SB, MB, XT, VS and KW are employees and stockholders of AstraZeneca. RP-F reports honoraria (paid to employer) from AstraZeneca, Boehringer-Lilly, Akebia, Bayer, GSK, and Novo Nordisk for participation in Advisory Boards and educational activities; consulting fees and scientific leadership in clinical trials from the George Clinical; research grants from Fresenius Medical Care, and National Council for Scientific and Technological Development. RP-F is employed by Arbor Research Collaborative for health, who runs the DOPPS studies. Global support for the ongoing DOPPS Programs is provided without restriction on publications by a variety of funders. Funding is provided to Arbor Research Collaborative for Health and not directly to RP-F. For details see https://www.dopps.org/AboutUs/Support.aspx. HJLH has received research grants from AstraZeneca, Boehringer Ingelheim, Janssen, and NovoNordisk (payments to his employer). His institution received honoraria from AstraZeneca, Bayer, Boehringer Ingelheim, Chinook, CSL Behring, Dimerix, Eli-Lilly, Janssen, Gilead, Merck, Novartis, NovoNordisk, and Travere Therapeutics for participation in steering committees of clinical trials and consultancies.

## Supplementary Material

Supplementary File (PDF) **Supplementary Methods. Supplementary References. Figure S1.***IL-33* mRNA levels in an independent cohort of patients with advanced DKD and correlation with eGFR. **Figure S2.** IL-33 protein expression in biopsies from healthy donors and patients with DKD. **Figure S3.***IL-33* gene signature in the tubulointerstitium. **Figure S4.** Renal *IL-33* mRNA and IL-33 protein expression in a T1D DKD preclinical mouse model. **Figure S5.** Anti-ST2 antibody plasma exposure. **Figure S6.** Blood cell counts. **Figure S7.** Effects on (A) body weight, (B) fed blood glucose, (C) percentage hemoglobin A1c, and (D) urine volume, following treatment with an anti-ST2 antibody. **Figure S8.** Cell type-specific expression of IL-33 in the kidney by single-nucleus RNA sequencing. **Figure S9.** Intracellular IL-33 protein expression in response to inflammatory stimuli in (A) mesangial cells and (B) proximal tubule epithelial cells. **Figure S10.** IL-33 induces an inflammatory response in glomerular microvascular endothelial cells. **Figure S11.** IL-33 induces a modest or no inflammatory response through ST2 signaling in (A–C) mesangial cells and in (D–G) RPTEC. **Figure S12.** Tozorakimab blocks IL-33–induced inflammatory response in glomerular microvascular endothelial cells. **Figure S13.** Ultrasensitive, selective assays for IL-33 forms. **Figure S14.** Assessment of urine inflammatory biomarkers in patients with DKD. **Figure S15.** Distribution of baseline hsCRP. **Figure S16.** Distribution of baseline eosinophils. **Figure S17.** Histogram of baseline (A) CCL2, (B) TNFR1, and (C) TNFR2. **Figure S18.** Distribution of baseline (A) CCL2, (B) TNFR1, and (C) TNFR2. **Figure S19.** Histogram of log-transformed baseline (A) CCL2, (B) TNFR1, and (C) TNFR2. **Figure S20.** Distribution of baseline log-transformed (A) CCL2, (B) TNFR1, and (C) TNFR2. **Figure S21.** Distribution of baseline UACR. **Figure S22.** Distribution of log-transformed baseline UACR. **Figure S23.** Correlation of CCL2, TNFR1, and TNFR2 with UACR. **Table S1.** Reference cohorts for exploratory analysis. **Table S2.** Number of human samples used for IL-33 gene signature analyses. **CONSORT Statement.**
