# Supplementary material for: Inhibition of Interleukin-33 to Reduce Glomerular Endothelial Inflammation in Diabetic Kidney Disease
Source: Kidney Int Rep. 2024 Mar 18;9(6):1876–91. doi: 10.1016/j.ekir.2024.03.009 (PMC11184260; doi:10.1016/j.ekir.2024.03.009)
Supplement: Supplementary File (PDF) — Supplementary Methods. Supplementary References. Figure S1.IL-33 mRNA levels in an independent cohort of patients with advanced DKD and correlation with eGFR. Figure S2. IL-33 protein expression in biopsies from healthy donors and patients with DKD. Figure S3.IL-33 gene signature in the tubulointerstitium. Figure S4. Renal IL-33 mRNA and IL-33 protein expression in a T1D DKD preclinical mouse model. Figure S5. Anti-ST2 antibody plasma exposure. Figure S6. Blood cell counts. Figure S7. Effects on (A) body weight, (B) fed blood glucose, (C) percentage hemoglobin A1c, and (D) urine volume, following treatment with an anti-ST2 antibody. Figure S8. Cell type-specific expression of IL-33 in the kidney by single-nucleus RNA sequencing. Figure S9. Intracellular IL-33 protein expression in response to inflammatory stimuli in (A) mesangial cells and (B) proximal tubule epithelial cells. Figure S10. IL-33 induces an inflammatory response in glomerular microvascular endothelial cells. Figure S11. IL-33 induces a modest or no inflammatory response through ST2 signaling in (A–C) mesangial cells and in (D–G) RPTEC. Figure S12. Tozorakimab blocks IL-33–induced inflammatory response in glomerular microvascular endothelial cells. Figure S13. Ultrasensitive, selective assays for IL-33 forms. Figure S14. Assessment of urine inflammatory biomarkers in patients with DKD. Figure S15. Distribution of baseline hsCRP. Figure S16. Distribution of baseline eosinophils. Figure S17. Histogram of baseline (A) CCL2, (B) TNFR1, and (C) TNFR2. Figure S18. Distribution of baseline (A) CCL2, (B) TNFR1, and (C) TNFR2. Figure S19. Histogram of log-transformed baseline (A) CCL2, (B) TNFR1, and (C) TNFR2. Figure S20. Distribution of baseline log-transformed (A) CCL2, (B) TNFR1, and (C) TNFR2. Figure S21. Distribution of baseline UACR. Figure S22. Distribution of log-transformed baseline UACR. Figure S23. Correlation of CCL2, TNFR1, and TNFR2 with UACR. Table S1. Reference cohorts for exploratory analysis. [file mmc1.pdf]

Inhibition of IL-33 to reduce glomerular endothelial inflammation in DKD

Alexis Hofherr,[1,,#] Elena Liarte Marin,[2,*] Barbara Musial,[2] Asha Seth,[2] Tim Slidel,[3] James Conway,[4] David Baker,[5] Pernille B.L. Hansen,[2] Benjamin Challis,[6] Stefano Bartesaghi,[6] Maria Bhat,[6] Roberto Pecoits-Filho,[7,8,9] Xiao Tu,[10] Viknesh Selvarajah,[11] Kevin Woollard,[2,#] and Hiddo JL Heerspink[9,12]*

* These authors contributed equally to this work.

[#] Joint corresponding authors:

alexis.hofherr@astrazeneca.com
kevin.woollard@astrazeneca.com

[1]Research and Early Clinical Development, Cardiovascular, Renal and Metabolism (CVRM), BioPharmaceuticals R&D, AstraZeneca, Gothenburg, Sweden

[2]Bioscience Renal, Research and Early Development, Cardiovascular, Renal and Metabolism (CVRM), BioPharmaceuticals R&D, AstraZeneca, Cambridge, UK

[3]Bioinformatics, Oncology R&D, AstraZeneca, Cambridge, UK

[4]Bioinformatics, Oncology R&D, AstraZeneca, Gaithersburg, MD, USA

[5]Bioscience Metabolism, Research and Early Development, Cardiovascular, Renal and Metabolism (CVRM), BioPharmaceuticals R&D, AstraZeneca, Cambridge, UK

[6]Translational Science & Experimental Medicine, Research and Early Clinical Development, Cardiovascular, Renal and Metabolism (CVRM), BioPharmaceuticals R&D, AstraZeneca, Gothenburg, Sweden

[7]Arbor Research Collaborative for Health, Ann Arbor, MI, USA

[8]School of Medicine, Pontificia Universidade de Catolica do Parana, Curitiba, Brazil

[9]The George Institute for Global Health, University of New South Wales Sydney, Sydney, NSW, Australia

[10]Research and Early Clinical Development, Cardiovascular, Renal and Metabolism (CVRM), BioPharmaceuticals R&D, AstraZeneca, Gaithersburg, MD, USA

[11]Research and Early Clinical Development, Cardiovascular, Renal and Metabolism (CVRM), BioPharmaceuticals R&D, AstraZeneca, Cambridge, UK

[12]Department of Clinical Pharmacy and Pharmacology, University of Groningen, University Medical Center Groningen, Groningen, Netherlands

Table of Content

SUPPLEMENTAL METHODS **4**

Evaluation of Kidney Biopsies 4

Targeted Analysis of Inflammatory Gene Expression *4*

Kidney Expression of IL-33 in Patients with CKD *4*

Immunohistochemistry 4

mRNA-based Assessment of IL-33 Pathway Signature 5

Mouse Models and Analysis 5

db/db Uninephrectomy *5*

Body Mass, Treatment Exposure, Creatinine, Albuminuria, and HbA$_{1c}$ *6*

Histological Analysis *6*

Streptozotocin (STZ) High-fat Diet *6*

IL-33 Detection *7*

In Vitro Analysis of IL-33 Signaling 7

Cell Culture *7*

Recombinant IL-33 Protein Production *8*

MAP Kinase Assay *8*

NF-κB Translocation *8*

Cytokine Detection *9*

Proliferation Assays *9*

qPCR *10*

Reference Cohorts for Exploratory Biomarker Analysis 10

FRONTIER-1: Tozorakimab in Adults with DKD 10

Overview *10*

Ethics and Conduct *11*

Participants *11*

Trial Procedures *11*

Outcomes *12*

Biomarker Measurement *13*

Linear Mixed-effect Model with Trend *13*

SUPPLEMENTAL TABLES AND FIGURES **14**

Table S1. Reference cohorts for exploratory analysis. 14

Table S2. Number of human samples used for IL-33 gene signature analyses 14

Figure S1. *IL-33* mRNA levels in an independent cohort of patients with advanced DKD and correlation with eGFR. 15

Figure S2. IL-33 protein expression in biopsies from healthy donors and patients with DKD. 16

Figure S3. *IL-33* gene signature in the tubulointerstitium. 17

Figure S4. Renal IL-33 mRNA and IL-33 protein expression in a T1D DKD pre-clinical mouse model. 18

Figure S5. Anti-ST2 antibody plasma exposure. 19

Figure S6. Blood cell counts. 20

Figure S7. Effects on (A) body weight, (B) fed blood glucose, (C) percentage hemoglobin A1c, and (D) urine volume, following treatment with an anti-ST2 antibody. 21

Figure S8. Cell type-specific expression of IL-33 by single-nucleus RNA sequencing. 22

Figure S9. Intracellular IL-33 protein expression in response to inflammatory stimuli in (A) mesangial cells and (B) proximal tubule epithelial cells. 23

Figure S10. IL-33 induces an inflammatory response in glomerular microvascular endothelial cells. 24

Figure S11. IL-33 induces a modest or no inflammatory response through ST2 signaling in (A–C) mesangial cells and in (D–G) RPTEC. 25

Figure S12. Tozorakimab blocks IL-33 induced inflammatory response in glomerular microvascular endothelial cells. 26
Figure S13. Ultra-sensitive, selective assays for IL-33 forms. 27
Figure S14. Assessment of urine inflammatory biomarkers in patients with DKD. 28
Figure S15. Distribution of baseline hsCRP. 29
Figure S16. Distribution of baseline eosinophils. 30
Figure S17. Histogram of baseline (A) CCL2, (B) TNFR1, and (C) TNFR2. 31
Figure S18. Distribution of baseline (A) CCL2, (B) TNFR1, and (C) TNFR2. 32
Figure S19. Histogram of log-transformed baseline (A) CCL2, (B) TNFR1, and (C) TNFR2. 33
Figure S20. Distribution of baseline log-transformed (A) CCL2, (B) TNFR1, and (C) TNFR2. 34
Figure S21. Distribution of baseline UACR. 35
Figure S22. Distribution of log-transformed baseline UACR. 36
Figure S23. Correlation of CCL2, TNFR1, and TNFR2 with UACR. 37

SUPPLEMENTAL REFERENCES 38

CONSORT STATEMENT 39

SUPPLEMENTAL METHODS

Evaluation of Kidney Biopsies

Targeted Analysis of Inflammatory Gene Expression

Data from the Karolinska Institute/Sahlgrenska University Hospital (KI/SU) human kidney cohort were used to analyze inflammatory marker expression in patients with DKD. Briefly, RNA sequencing was performed on paired, microdissected glomerular and tubulointerstitial tissue from patients diagnosed with DKD (*n*=19; 15 males; median age, 61 [range: 30–85] years; CKD stages 1–4) and living kidney donors (*n*=20; 12 males; median age: 56 [30–70] years), as previously described.[21] The *P* values were adjusted for multiple testing using the Benjamini–Hochberg method to identify pro-inflammatory cytokines that were significantly differentially expressed in each tissue compartment. The threshold for significance was set at an adjusted *P* value of <0.05.

Kidney Expression of IL-33 in Patients with CKD

Human kidney samples were processed according to the European Renal cDNA Bank (ERCB) protocol as previously described.[22,23] IL-33 expression in biopsies from living kidney donors was compared with IL-33 expression in biopsies from patients with various CKD etiologies.

Immunohistochemistry

Fixed 3–4 µm human kidney sections were stained on an automated immunohistochemistry robot (Flatbed Autostainer Plus, Dako; Santa Clara, CA, USA). The EnVision FLEX High pH (Link) detection kit (Dako K8000) was used. Paraffin sections underwent heat-induced epitope retrieval in Tris/EDTA buffer, pH 9 (K8004, Dako) in a microwave oven. Endogenous peroxidase activity was blocked with EnVision FLEX Peroxidase-Blocking Reagent for 5 min at room temperature. For immunostaining, a goat anti-human IL-33 antibody (AF3625, R&D Systems; Minneapolis, MN, USA) was incubated for 30 min at room temperature followed by polymer/horseradish peroxidase-linked secondary antibody

(Dako) for a further 30 min and transferred to DAB solution for 4 min. Sections were stained with Mayer's hematoxylin, dehydrated with ethanol, and mounted under cover slips. Analysis was done by using the Aperio ePathology digital image analysis software.

mRNA-based Assessment of IL-33 Pathway Signature

Human renal biopsies from the ERCB cohort were microdissected for glomeruli and tubulointerstitial compartment separation and profiled by microarray analysis as described previously.[22,24] Gene Set Variation Analysis (GSVA) was performed in this dataset and a signature score (scaled as $-1 \leq x \leq 1$) was calculated based on expression data from a predefined gene set (*IL-33*, *interleukin 1 receptor-like 1* [*ST2*], *interleukin 1 receptor accessory protein* [*IL1RAP*], *TNF receptor-associated factor* 6 [*TRAF6*], *myeloid differentiation primary response 88* [*MYD88*], and *interleukin-1 receptor-associated kinase 4 [IRAK-4]*).[25]

Mouse Models and Analysis

db/db Uninephrectomy

5-week-old male *db/db* mice (BKS.Cg-Dock7m +/+ Leprdb/J homozygotes; Charles River, Italy) were housed in the Medimmune Biological Sciences Unit (Cambridge, UK) on a 12 h light/dark cycle, with ad libitum access to water and Purina 5008 chow. All animal protocols and procedures complied with the guidelines and regulations of a UK Home Office Project Licence, which had been reviewed and approved by the local Animal Welfare and Ethical Review Body (AWERB).

Mice were subjected to right nephrectomy under anesthesia, with post-operative analgesia, at 7 weeks to hasten the development of DKD. Mice were randomized by urine albumin-to-creatinine ratio (UACR) at 10 weeks of age. Anti-ST2 (ZY0NP0_E06) mIgG1 (*n*=13) or an isotype control antibody (n=14) was administered intraperitoneally from 11–16 weeks of age. All treatments were

administered at a dose of 10 mg/kg three times per week. Urine was collected at 10, 13, and 15 weeks of age. Terminal tissues were collected at 16 weeks of age.

Body Mass, Treatment Exposure, Creatinine, Albuminuria, and HbA$_{1c}$

Body mass was monitored once every 2/3 days and other parameters were measured at 10 (baseline), 13, and/or 15 weeks of age. Blood samples were collected from the tail vein using a K2EDTA microvette. To collect urine samples, mice were housed in metabolic cages with free access to food and water for an 18-h period. To reduce evaporation, the urine collectors were placed in a pre-chilled plastic thermoblock (Tecniplast; Buguggiate VA, Italy). Fed glucose was measured by taking a drop of blood from a tail prick and applying it to a Nova StatStrip glucose meter (Data Sciences International; St. Paul, MN, USA). Antibody plasma exposure was determined by an enzyme-linked immunosorbent assay (ELISA) developed in house that detected anti-mouse ST2 (ZYONP0-F06). Urinary creatinine and albumin concentrations and percentage hemoglobin A1c (HbA1c) were measured using the Cobas C111 analyzer (Roche; Basel, Switzerland).

Histological Analysis

Kidneys were collected at study termination, fixed in 10% neutral buffered formalin for 48 h and paraffin embedded. Fixed 3- to 4-µm sections were stained with periodic acid–Schiff (PAS) stain to examine mesangial expansion and glomerular damage. 30–40 glomeruli were scored per kidney from the upper half of the section throughout the full thickness of the cortex. Mice were scored 0–4, based on extent of PAS-positive mesangial expansion, scarcity of nuclei, and decrease in capillary luminal spaces.

Streptozotocin (STZ) High-fat Diet

This study was conducted by RenaSci Limited (Nottingham, UK) in accordance with the project license and Animals (Scientific Procedures) Act 1986. C57bl6/J (4–6 weeks of age, >15 g) mice were sourced from Charles River Laboratories (Wilmington, MA, USA) and RD12492 (a 60% high-fat diet)

from Research Diets (New Brunswick, NJ, USA). Upon arrival, mice were introduced to the high-fat diet, which commenced 3 weeks prior to diabetes induction and persisted through the study. Streptozotocin (STZ), a cytotoxic compound targeting pancreatic islet β-cells, was used to abolish insulin secretion and induce hyperglycemia. For a period of 5 consecutive days, STZ or a vehicle control was administered intraperitoneally at repeat low doses (50 mg/kg). The study was concluded 16 weeks after the initiation of STZ dosing, and the kidneys were collected for RNA and protein analysis of IL-33.

IL-33 Detection

Kidney lysates were prepared by homogenization of kidney tissue in 0.05% Triton x-100 in phosphate-buffered saline buffer with added protease inhibitors. Mouse IL-33 protein levels from kidney homogenates were quantified using the MILLIPLEX Mouse TH17 Magnetic Bead Panel (MTH17MAG-47K, Merck Millipore; Burlington, MA, USA), according to the manufacturer's instructions.

Cells were seeded into 6-well plates at 2×10^6 cells/well and were incubated with indicated agonists, or left unstimulated, for 24 h. Human IL-33 protein levels from cell lysates were measured using a U-PLEX Human IL-33 Assay (K151WFK, Meso Scale Discovery; Rockville, MD, USA).

In Vitro Analysis of IL-33 Signaling

Cell Culture

Human glomerular microvascular endothelial cells (HGMECs; ACBRI 128, Cell Systems; Kirkland, WA, USA) were cultured in Complete Classic Medium (4Z0-500, Cell Systems) supplemented with CultureBoost (4CB-500, Cell Systems) and following the coating procedures using Attachment Factor (4Z0-201/4Z0-210, Cell Systems), according to the manufacturer's instructions. Human kidney mesangial cells (3031, Novabiosis; Durham, NC, USA) were cultured in Mesangial Cell Medium (4201,

ScienCell; Carlsbad, CA, USA) containing 2% fetal bovine serum (FBS; 0010, ScienCell), 1% mesangial cell growth supplement (MsCGS; 4252, ScienCell), and 1% penicillin/streptomycin solution (P/S; 0503, ScienCell), according to the manufacturer's instructions. Human Renal Proximal Tubule Epithelial Cells (PTECs; CC-2553, Lonza; Basel, Switzerland) were cultured in REBM Basal Medium (CC-3191, Lonza) and REGM SingleQuots supplements (CC-4127, Lonza), according to the manufacturer's instructions. Cells were grown to confluence and harvested with accutase (L11-007, PAA Laboratories; Pasching, Austria) for use in the bioassays described below.

Recombinant IL-33 Protein Production

Cloning, expression, and purification of wild-type (WT) human IL-33 (aa 112–270) and a variant with all four cysteine residues mutated to serine (IL-33C>S) that is resistant to oxidation were synthesized as described previously.[26]

MAP Kinase Assay

Cells were seeded in 24-well plates at 5×10^4 cells/well and treated with 30 ng/mL of IL-33 with or without 1 µg/mL of IL-33-neutralizing antibody (tozorakimab) or human IgG1 isotype control, or left untreated, and incubated at 37°C for 30 min. Cells were washed and lysed with corresponding kit buffers. Protein extracts were centrifuged at 14,000 RPM at 4°C and protein concentrations were determined using the BCA Protein Assay Kit (23225, Thermo; Waltham, MA, USA). Phosphorylated mitogen-activated protein kinases (MAPK), p38, and c-Jun N-terminal kinase (JNK) were detected using a Meso Scale diagnostic assay (Phospho-p38 kit, K150CYD, Meso Scale Discovery; Phospho-JNK kit, K150CUD, Meso Scale Discovery, respectively), according to the manufacturer's instructions.

NF-κB Translocation

Cells were seeded into 96-well plates at 1×10^4 cells/well and stimulated with a full dose range of IL-33 or IL-1β (positive control; 200-01B, Peprotech; Rocky Hill, NJ, USA), or stimulated with 30 ng/mL of IL-33 or IL-1β together in the presence of 1 µg/mL of tozorakimab or human IgG1 isotype control,

or left untreated, and then incubated at 37°C for 24 h. NF-κB translocation to the nucleus was measured by immunofluorescence.[27] Cells were fixed with 10% neutral-buffered formalin solution (HT5011, Sigma; St. Louis, MO, USA), permeabilized with PBS/0.1% Triton X-100 and stained with NF-κB p65 (PA5-16545, Thermo), goat anti-rabbit IgG Alexa Flour 488 (35552, Invitrogen; Waltham, MA, USA), and Hoechst 33342 (62249, Thermo), according to the manufacturer's instructions. Plates were read and analyzed for nuclear NF-κB p65 translocation using a Cytation 5 Cell Imaging Reader.

Cytokine Detection

Cells were seeded into 24-well plates at 1×10^5 cells/well. In some assays, cells were stimulated with a full dose range of IL-33 and incubated at 37°C for 24 h. In other assays, cells were treated with 30 ng/mL of IL-33 with or without 1 µg/mL of tozorakimab or human IgG1 isotype control, or left untreated, and incubated at 37°C for 24 h. Supernatants were collected for a detection of proinflammatory cytokines using a Meso Scale diagnostic assay (V-PLEX Proinflammatory Panel kit, K15049D or K15053D, Meso Scale Discovery; R-PLEX Human TNF-RI Assay, K1510VR, Meso Scale Discovery; and V-PLEX Human MCP-1 kit, K151NND, Meso Scale Discovery), according to the manufacturer's instructions.

Proliferation Assays

Cells were seeded in 96-well plates at 5×10^4 cells/well and incubated at 37°C for 16–18 h. Cells were then serum and cell growth supplement starved and incubated at 37°C for a further 24 hours. Cells were treated with increasing concentrations of IL-33 or platelet-derived growth factor-BB (100-14B-2UG, PeproTech) or epidermal growth factor (236-EG-200, R&D Systems) used as positive controls and incubated at 37°C for 18 h. Cells were pulsed with a 10 µM 5-ethynyl-2′-deoxyuridine (EdU) solution for a further 4 h. EdU incorporation was assessed using Amplex UltraRed reagent and the Click-iT EdU Proliferation Assay (C10499, Invitrogen) and fluorescence emission measurement, following the manufacturer's instructions.

qPCR

Cell pellets from confluent cells were lysed for RNA analysis. MagMax 96 Total RNA Isolation Kit (AM1830, Thermo) in the KingFisher Flex Purification System was used for RNA extraction following the manufacturer's protocol. For mouse kidney tissue, 2-mm biopsy punch was used to collect a sample from the renal cortex. The sample was transferred into RNAlater (#AM7020, Thermo), stored at 4°C for 24 h, and then the RNAlater was removed, and the sample frozen at –80°C. Tissue lysis and RNA extraction were performed using a TissueLyser II (Qiagen; Hilden, Germany) and the RNeasy Mini Kit (#74104, Qiagen), following the manufacturer's instructions.

For reverse transcription quantitative PCR (RT-qPCR), TaqMan RNA-to-CT 1-Step Kit (4392938, Thermo) was used together with an *IL-33* FAM probe (Hs04931857_m1, Thermo) or *ST2* (Hs00249384_m1, Thermo) and a *GAPDH* VIC probe (Hs99999905_m1, Thermo) or a murine *il-33* FAM probe (Mm00505403_m1, Thermo) and a murine *gapdh* VIC probe (Mm99999915_g1, Thermo). The mix was added to a MicroAmp EnduraPlate (4483273, Thermo). Plates were sealed and briefly centrifuged before analysis using a QuantStudio 12 Flex Real-Time PCR system (Thermo). The ΔΔCT method was used to calculate the relative gene expression of samples.

Reference Cohorts for Exploratory Biomarker Analysis

Supplemental Table 1 describes the reference cohorts used in the exploratory biomarker analysis.

FRONTIER-1: Tozorakimab in Adults with DKD

Overview

FRONTIER-1 is a randomized, double-blind, placebo-controlled, multicenter, phase 2b, dose-finding clinical trial to assess the efficacy, safety, pharmacokinetics, and immunogenicity of tozorakimab in adults with diabetic kidney disease (DKD). Study completion was in May 2023 with full results expected in spring 2024.

Ethics and Conduct

FRONTIER-1 was sponsored by AstraZeneca and started in December 2019. The trial was conducted at 97 sites in seven countries. FRONTIER-1 is registered at ClinicalTrials.gov (NCT04170543) and conforms to the principles of the Declaration of Helsinki, the International Conference on Harmonisation Good Clinical Practice, and applicable regulatory requirements. Central or local ethics committees reviewed and approved the trial protocol and participants provided written informed consent before trial enrollment. The safety of participants was overseen by an independent data review monitoring committee.

Participants

Eligible patients were adults with type 2 diabetes (T2D) with an estimated glomerular filtration rate (eGFR) of 25–75 mL/min/1.73 m^2 and a urinary albumin-to-creatinine ratio (UACR) of 100–3000 mg/g. Participants were receiving a stable dose of an angiotensin-converting enzyme inhibitors (ACEi) or angiotensin II receptor blocker (ARB) for at least 6 weeks before screening. Participants with a documented intolerance to ACEi or ARBs were also eligible. Patients taking sodium–glucose cotransporter 2 inhibitors (SGLT2is) had to be on a stable dose for at least 4 weeks before randomization. Key exclusion criteria were a diagnosis of chronic kidney disease (CKD) other than DKD, hemoglobin A1c (HbA1c) >10.5%, serum potassium >5.5 mmol/L that could not be adjusted by appropriate management, and a history of clinically significant heart disease.

Trial Procedures

Approximately 565 patients were planned to be randomized (95:95:95:140:140) to receive four doses of tozorakimab, or volume-matched placebo dosed subcutaneously over a 168-day (24-week) treatment period. All participants received 10 mg dapagliflozin once-daily starting on Day 85 until Day 168 to assess the interaction between tozorakimab and SGLT2 inhibition, followed by a 70-day (10-week) follow-up period. All participants taking an SGLT2i at baseline were switched to 10 mg

dapagliflozin on Day 85. Participants and all trial personnel (except the members of the independent data review monitoring committee) will be blinded to treatment assignments throughout the trial.

After randomization, in-person trial visits were performed until Day 169. At each follow-up visit, vital signs were recorded, blood and urine samples were sent for laboratory assessment, and information on potential trial outcomes, adverse events (AEs), concomitant therapies, and adherence to the trial regimen were collected. Before trial completion, each participant underwent a final trial visit.

Outcomes

The primary outcome measure is the change from baseline to Day 169 (Week 24) in UACR compared with placebo to assess the effect of tozorakimab on albuminuria. Secondary outcomes are measures of safety and tolerability such as AEs, vitals, and electrocardiograms, as well as anti-drug antibody incidence throughout the study, and other efficacy measures such as tozorakimab serum concentrations and the proportion of participants with >30%, >40%, and >50% reduction in UACR. Exploratory objectives aim to assess IL-33 activity by evaluating change and percentage change from baseline for urine and/or serum/plasma biomarker of inflammation and fibrosis relevant to DKD progression.

The primary outcome will be assessed with a mixed model for repeated measures (MMRM) analysis of change from baseline to week 24 in UACR without multiplicity adjustment. The model is adjusted for fixed categorical effects of treatment, visit, and treatment-by-visit interaction, randomization stratification factors, and the continuous covariates of baseline log UACR and baseline log UACR-by-visit interaction. UACR will be log-transformed before entering the data in the MMRM analysis to alleviate the skewness of the data. Randomization stratification factors are with SGLT2i background or not, in Japan or the rest of the world.

Biomarker Measurement

The concentration of urine TNFR1/2 and CCL2 was measured using sandwich immunoassays. For both intra-assay and inter-assay precision, the quantifiable range of TNFR1/2 is 3.5–2,701.3 pg/mL. For CCL2, both intra-assay and inter-assay precision, the quantifiable range is 0.734–828.5 pg/mL (Meso Scale Discovery, V-PLEX Human MCP-1 Kit, K151-NND-2). The coefficient of variations was 88.52%, 68.0%, and 91.3% for TNFR1, TNFR2, and CCL2, respectively.

Linear Mixed-effect Model with Trend

The effectiveness of the therapy (Tr) with respect to the control treatment performance at inhibiting albuminuria progression will be assessed with a linear mixed-effect model.[S1,S2] The Yij, representing *i*th urine ACR (mg albumin/g creatinine) observed at *j*th assessment point (T), follows the linear growth model: Yij=a0i+a1i*Tj+eij, where a0i and a1i denote individual intercept and slope parameters, respectively, and eij~N(0,σ) represents model error. Both intercept and slope are assumed to express random effects: a0i=g00+g01*Tri+u0i, a1i=g10+g11*Tri+u1i, with u0i ~ N(0, σ0) and u1i ~ N(0, σ1). The parameters g00, g10, and g01, g11 represent the parameter's fixed effects; σ, and σ0, σ1 correspond to intra- and inter UACR variance, respectively. Treatment was defined as 'effective' if the albuminuria progression inhibition of the treated group compared with the control group was significantly different ($P<0.05$).

SUPPLEMENTAL TABLES AND FIGURES

Table S1. Reference cohorts for exploratory analysis.

Cohort	*N*=274	Description
Sun-MACRO[S3,S4]	80	Stage 2–4, fast progressors and stable patients
IMPROVE[S5,S6]	34	Stage 2–4, treatment with dapagliflozin vs SoC
CKD obs	100	Healthy to stage 4 (stage 2–4, *n*=75)
CITRINE[S7,S8]	60	Stage 3

Sun-MACRO. Two subgroups of participants from the Sun-MACRO study were identified. These were either fast progressors (*n*=30), defined as having an eGFR decline of >5 mL/min in 12 months, or participants with a stable eGFR (*n*=30), defined as having an eGFR change of <1 mL/min/year. **IMPROVE.** Overall, 34 participants with T2D from the IMPROVE study were included in these analyses. Participants had HbA1c levels between 55 and 100 mmoL/moL, and a first morning void UACR of ≥100 mg/g and <3500 mg/g. **CKD obs**. An observational study to investigate the immunopathophysiology of diabetic kidney disease. **CITRINE.** The CITRINE study enrolled 60 participants. Participants were adults with T2D, serum urate concentration ≥6.0 mg/dL, eGFR ≥30 mL/min/1.73 m^2, and a UACR of 30–3500 mg/g.

CKD, chronic kidney disease; eGFR, estimated glomerular filtration rate; HbA1c, hemoglobin A1c; obs, observational study; SoC, standard of care; T2D, type 2 diabetes; UACR, urinary albumin-to-creatinine ratio.

Table S2. Number of human samples used for IL-33 gene signature analyses.

Abbreviation	Sample	TI (*n*)	Glom (*n*)
LD	Living donor controls	46	48
TNx	Tumor nephrectomy	4	5
RPGN	Rapidly progressing glomerulonephritis	21	22
DN	Diabetic nephropathy	17	12
FSGS	Focal glomerular segmental sclerosis	13	18
HT	Hypertensive nephropathy	20	15
IgA	Immunoglobulin A nephropathy	25	27
MCD	Minimal change disease	13	14
MGN	Membranous glomerulonephritis	18	18
SLE	Systemic lupus erythematosus	32	32

Human samples from the ERCB were profiled by microarray analysis. Cohort consists of multiple CKD etiologies collected from both TI and the Glom in the kidney cortex.

ERCB, European Renal cDNA Bank; Glom, glomeruli; IL-33, interleukin-33; TI, tubulointerstitium.

Figure S1. *IL-33* mRNA levels in an independent cohort of patients with advanced DKD and correlation with eGFR.

(A) *IL-33* mRNA expression in patients with DKD relative to healthy control living donors in glomeruli (Wilcoxon, P=0.0076) and tubulointerstitium (Wilcoxon, P=0.0062) in the Woroniecka cohort.[S9] Boxes show the interquartile range, the middle horizontal line is the median, and whiskers indicate the minimum and maximum values. Individual data points are shown. **(B)** Correlation of *IL-33* mRNA expression with eGFR.

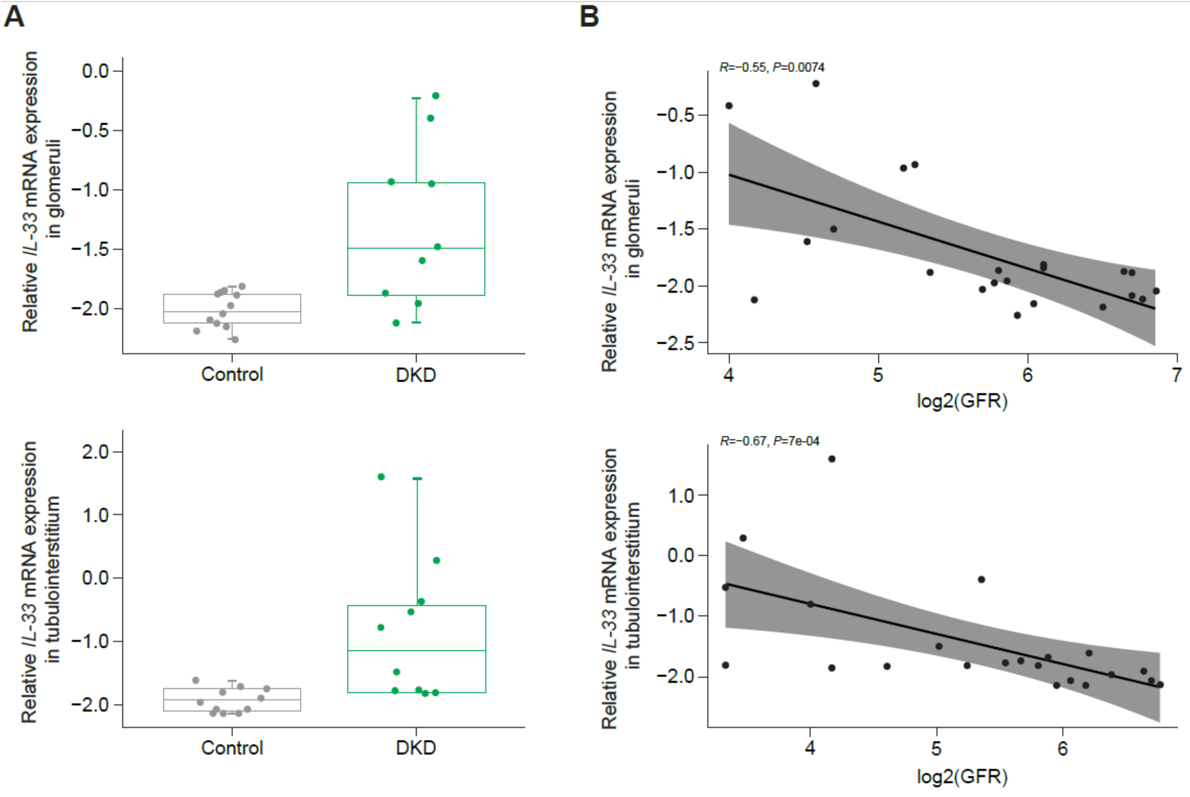

DKD, diabetic kidney disease; eGFR, estimated glomerular filtration rate; GFR, glomerular filtration rate; IL-33, interleukin-33.

Figure S2. IL-33 protein expression in biopsies from healthy donors and patients with DKD.

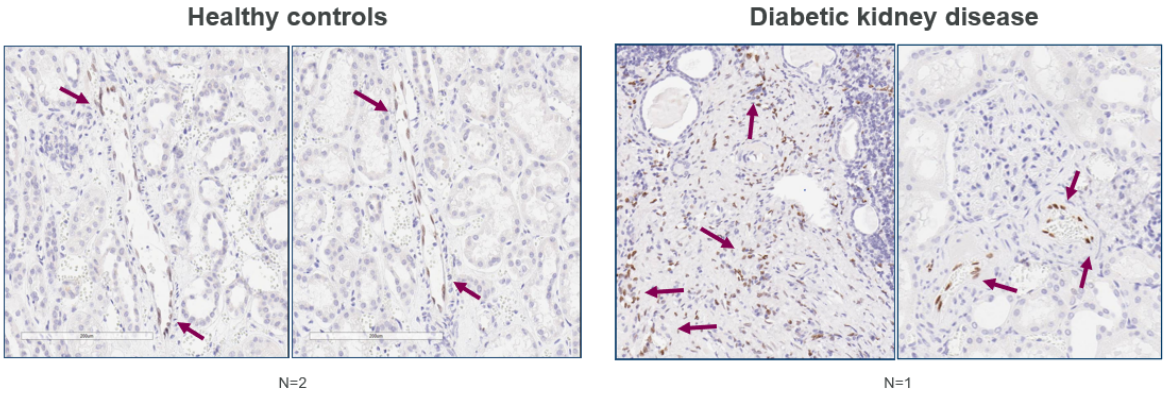

Arrows indicate IL-33 staining localization in the nuclei within endothelial cells of arterial and venous vessels. DKD sample shows immune cell infiltrates and inflammatory areas where IL-33-positivity is low to moderate. DKD, diabetic kidney disease; IL-33, interleukin-33.

Figure S3. *IL-33* gene signature in the tubulointerstitium.

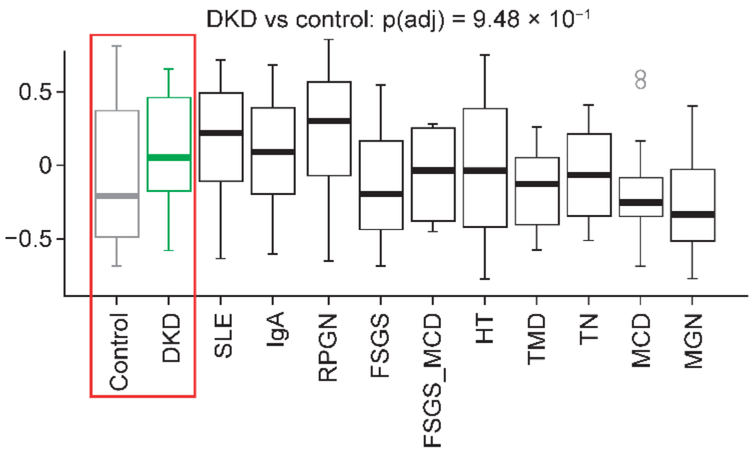

GSVA calculated signature score ($-1 \leq x \leq 1$) based on redefined *IL-33* signature gene set. The box plot shows the distribution of GSVA scores for each CKD condition. Boxes define the IQR; whiskers define, at maximum, 1.5×IQR. Outliers beyond 1.5×IQR are plotted individually. See **Table S2** for sample sizes. Adj, adjusted; CKD, chronic kidney disease; DKD, diabetic kidney disease; FSGS, focal glomerular segmental sclerosis; GSVA, gene set variation analysis; HT, hypertensive nephropathy; IgA, immunoglobulin A nephropathy; IL-33, interleukin-33; IQR, interquartile range; MCD, minimal change disease; MGN, membranous glomerulonephritis; RPGN, rapidly progressing glomerulonephritis; SLE, systemic lupus erythematosus; TMD, thin basement membrane disease; TN, tumor nephrectomy.

Figure S4. Renal IL-33 mRNA and IL-33 protein expression in a T1D DKD pre-clinical mouse model.

Renal **(A)** *IL-33* mRNA and **(B)** IL-33 protein expression in STZ-induced T1D mice at 16 weeks of age.

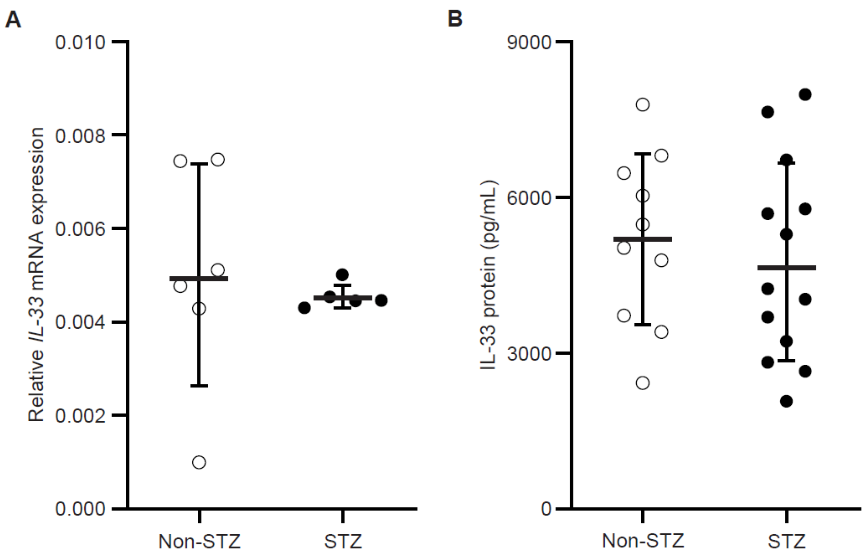

DKD, diabetic kidney disease; IL-33, interleukin-33; STZ, streptozotocin; T1D, type 1 diabetes.

Figure S5. Anti-ST2 antibody plasma exposure.

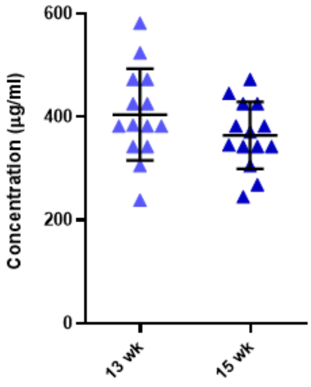

ST2, interleukin 1 receptor-like 1; wk, weeks.

Figure S6. Blood cell counts.

(A) Total cell counts for each subpopulation. **(B)** Percentage of cells of a given subpopulation.

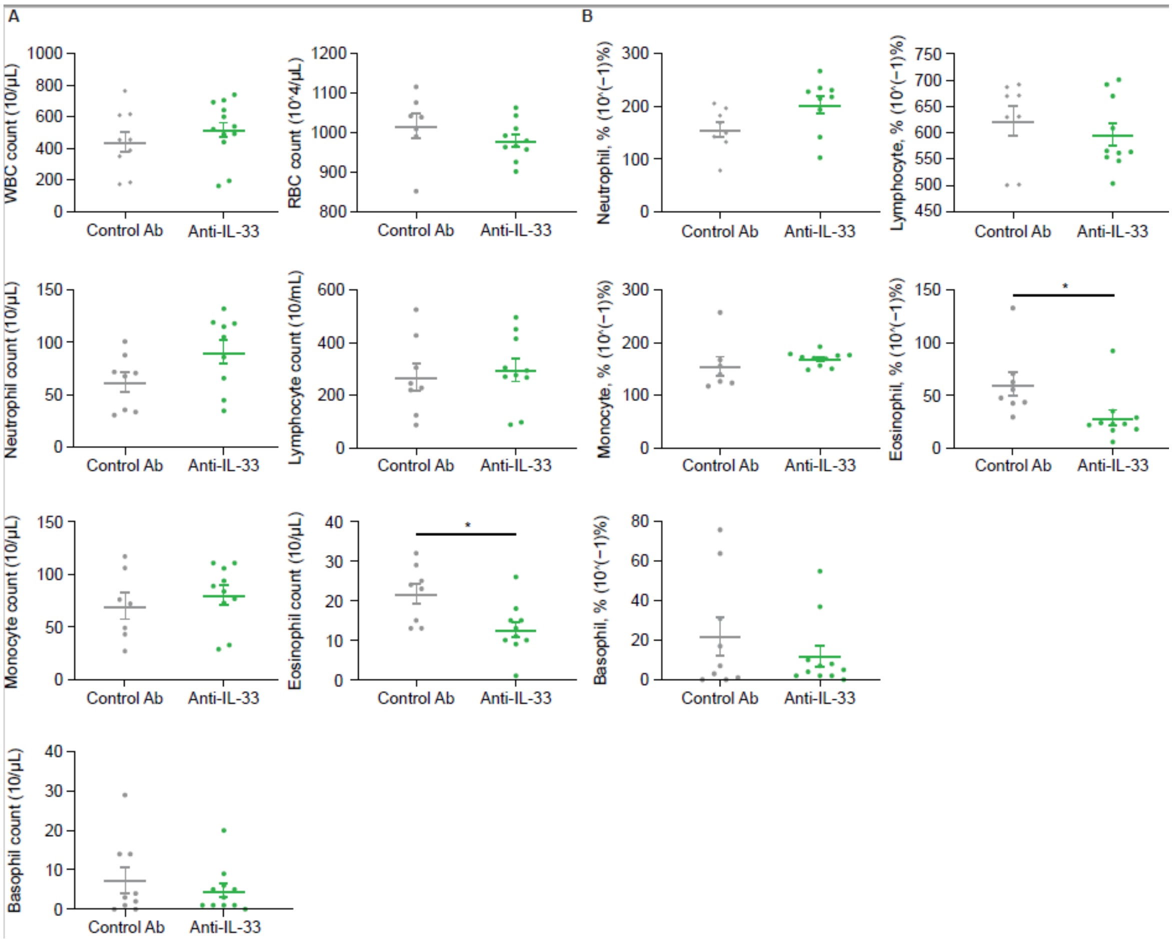

Ab, antibody; IL-33, interleukin-33; RBC, red blood cell; WBC, white blood cell.

Figure S7. Effects on **(A)** body weight, **(B)** fed blood glucose, **(C)** percentage hemoglobin A1c, and **(D)** urine volume, following treatment with an anti-ST2 antibody.

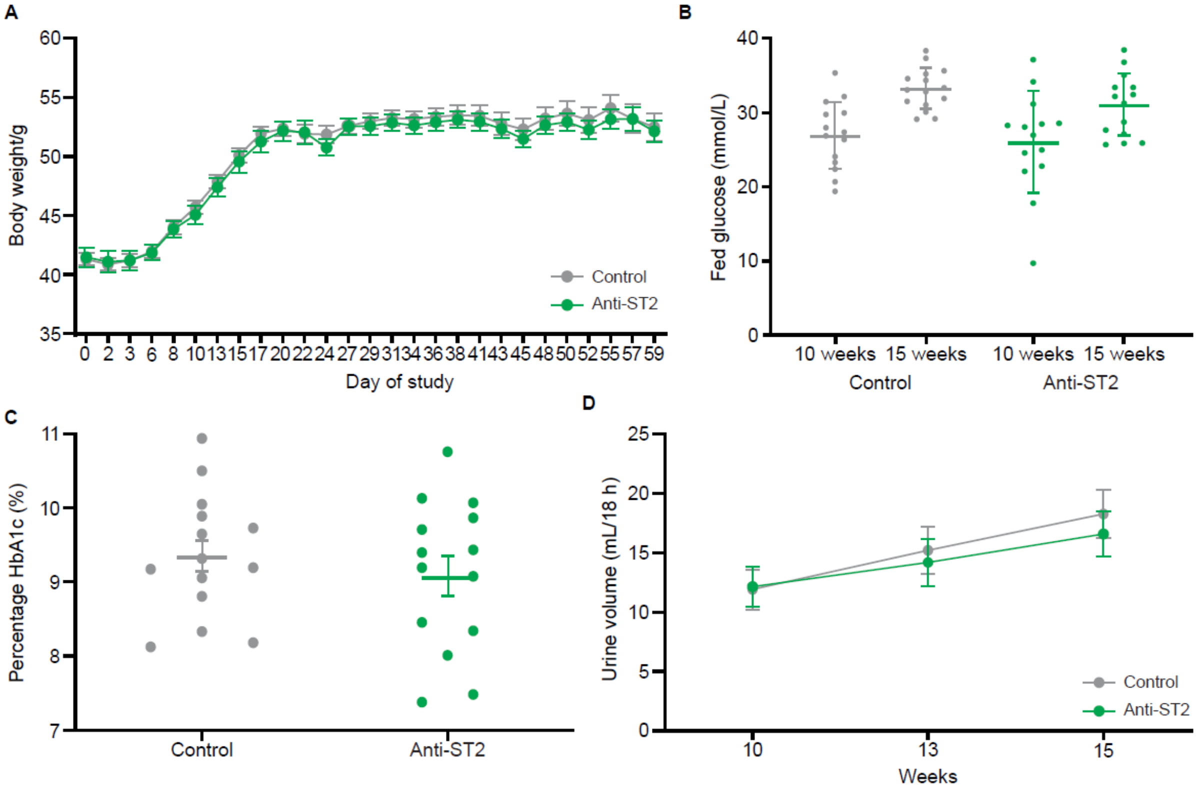

HbA1c, hemoglobin A1c; ST2, interleukin 1 receptor-like 1; wk, weeks.

Figure S8. Cell type-specific expression of IL-33 in the kidney by single-nucleus RNA sequencing.

Log-normalized UMI in the kidney of 3 patients with DKD and 3 non-diabetic controls (total = 23,980 transcriptomes).[S10]

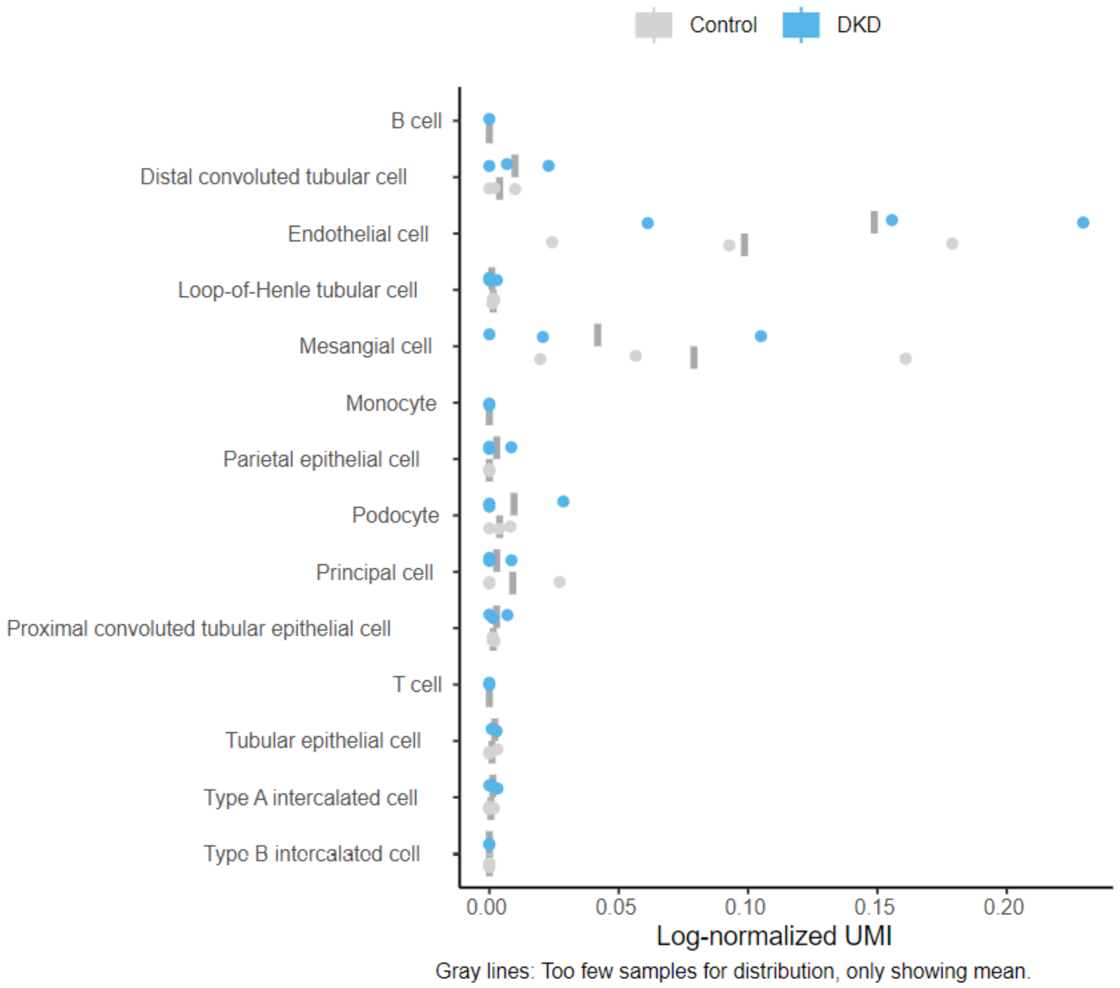

Figure S9. Intracellular IL-33 protein expression in response to inflammatory stimuli in **(A)** mesangial cells and **(B)** proximal tubule epithelial cells.

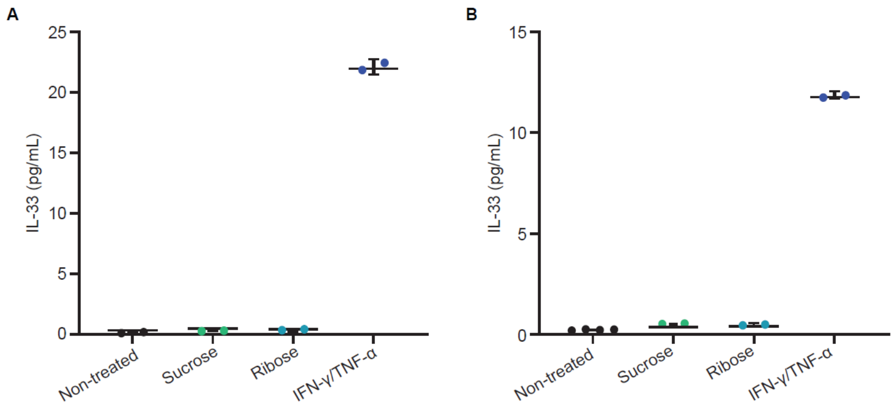

IFN-γ, interferon gamma; IL-33, interleukin-33; TNFα, tumor necrosis factor alpha.

Figure S10. IL-33 induces an inflammatory response in glomerular microvascular endothelial cells.

(A) Representative images of the nuclear translocation of NF-κB induced by IL-33 and IL-1β. **(B)** Dose-dependent release of pro-inflammatory cytokines in response to IL-33.

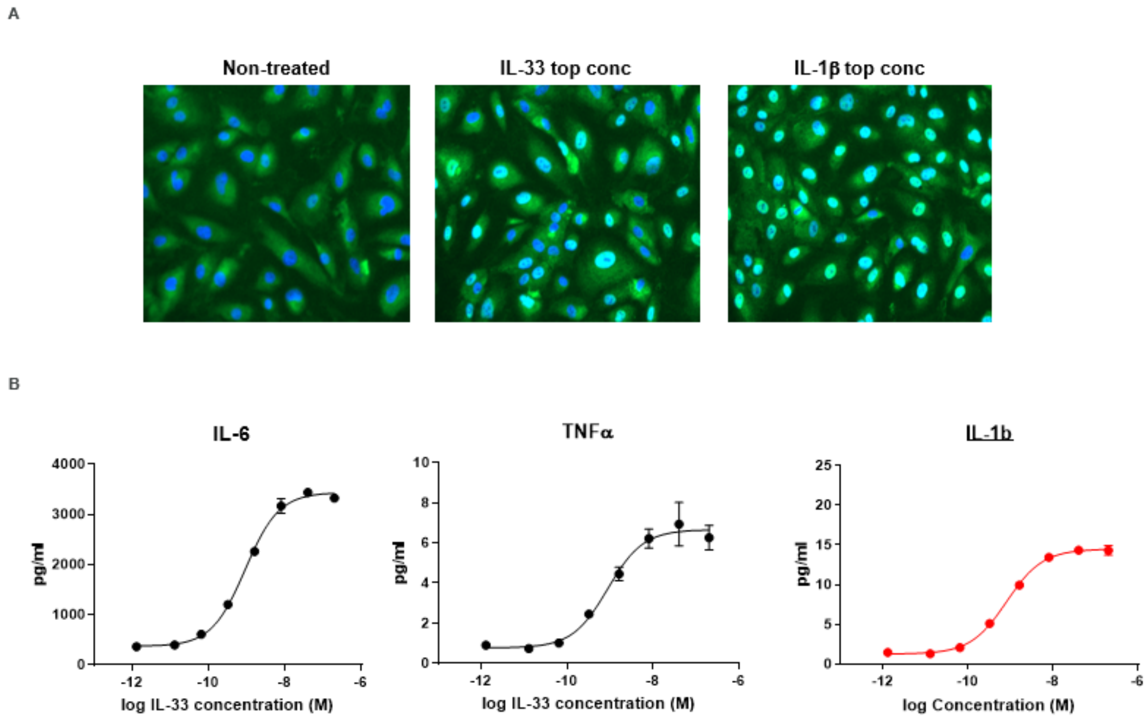

Conc, concentration; IL-1β , interleukin-1β; IL-6, interleukin-6; IL-33, interleukin-33; NF-κB, nuclear factor kappa-light-chain-enhancer of activated B cells; TNF-α, tumor necrosis factor alpha.

Figure S11. IL-33 induces a modest or no inflammatory response through ST2 signaling in **(A–C)** mesangial cells and in **(D–G)** RPTEC.

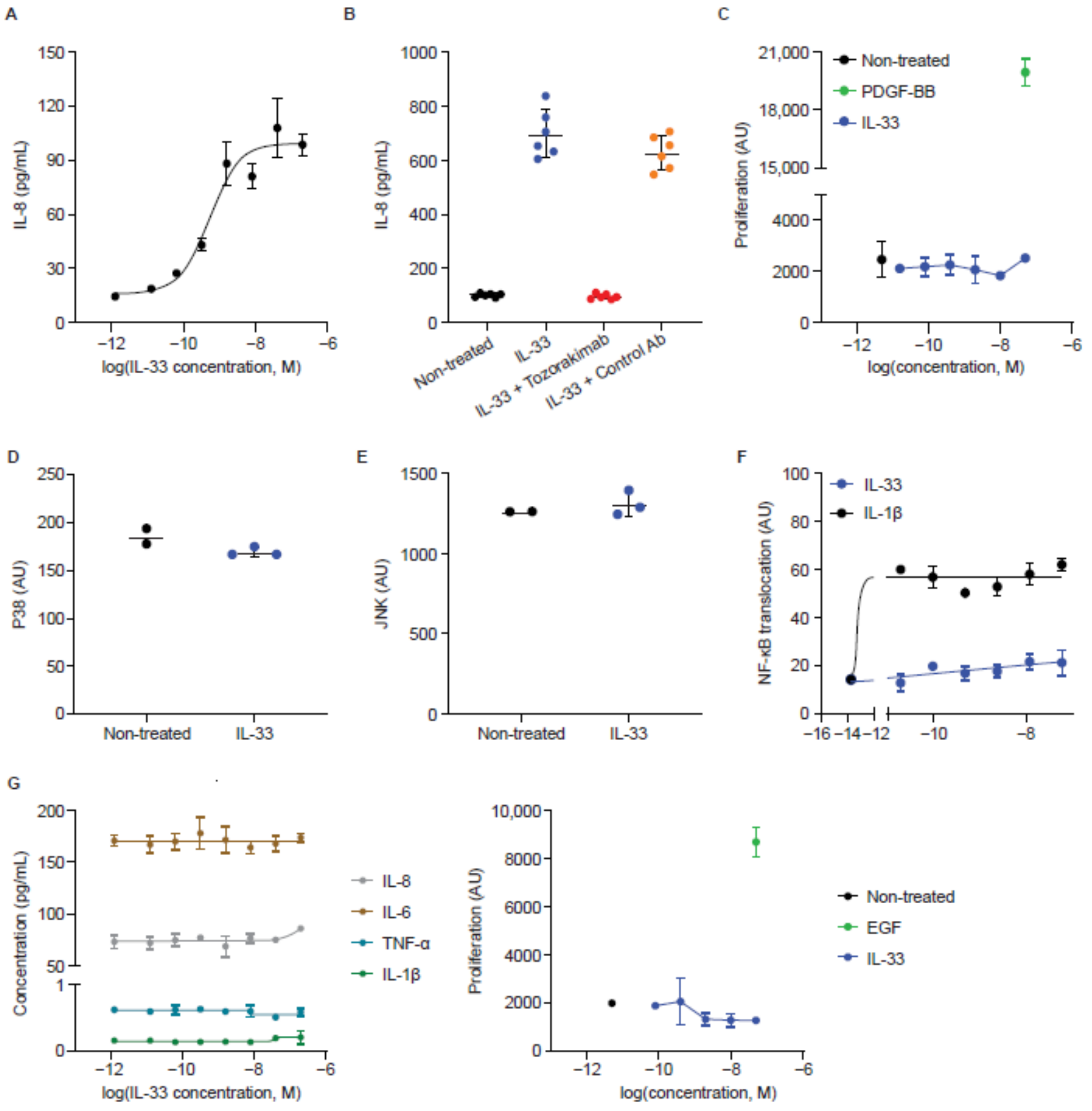

Ab, antibody; EGF, epidermal growth factor; IL-1β, interleukin-1β; IL-6, interleukin-6; IL-8, interleukin-8; IL-33, interleukin-33; PDGF-BB, platelet-derived growth factor-BB; RPTEC, renal proximal tubule epithelial cells; ST2, interleukin 1 receptor-like 1; TNF-α, tumor necrosis factor alpha.

Figure S12. Tozorakimab blocks IL-33 induced inflammatory response in glomerular microvascular endothelial cells.

(A) Representative images of the nuclear translocation of NF-kB induced by IL-33 and IL-1β in the presence of tozorakimab. **(B)** Tozorakimab inhibits IL-33 induced pro-inflammatory cytokine release.

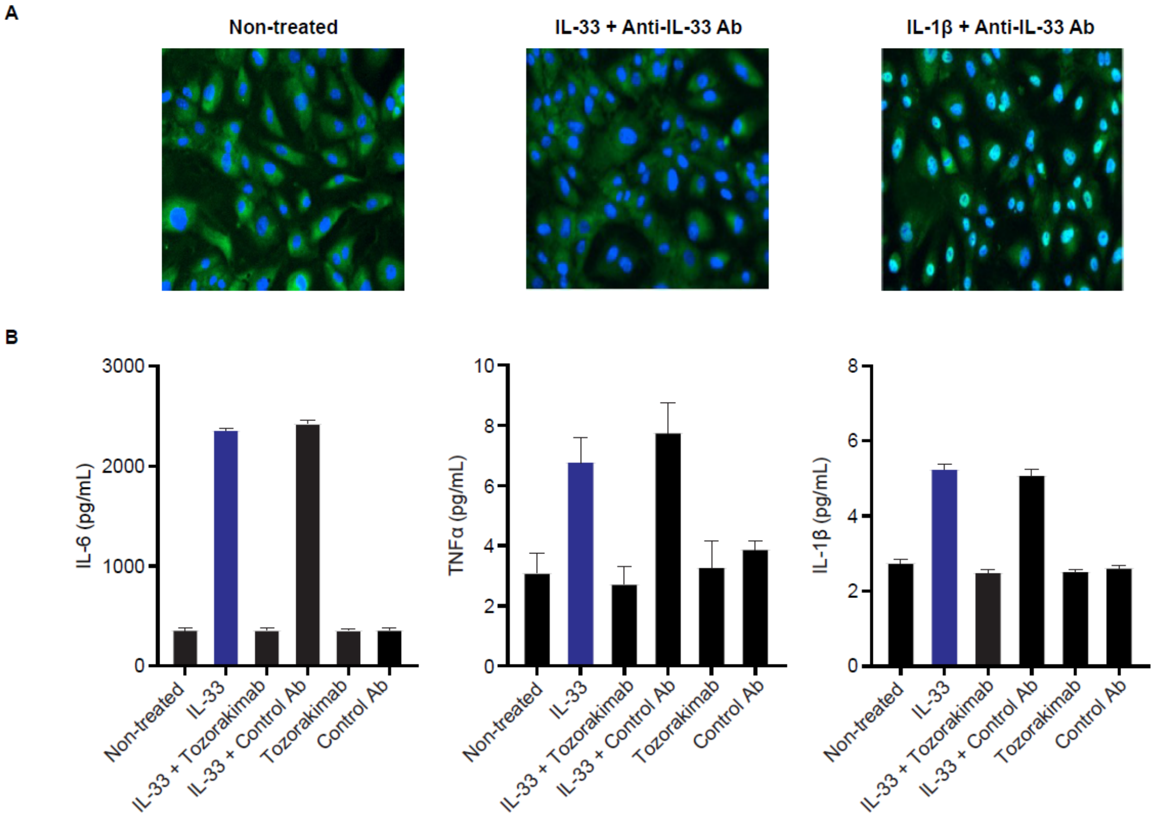

Ab, antibody; IL-1β, interleukin-1β; IL-6, interleukin 6; IL-33, interleukin-33; NF-kB, nuclear factor kappa-light-chain-enhancer of activated B cells.

Figure S13. Ultra-sensitive, selective assays for IL-33 forms.

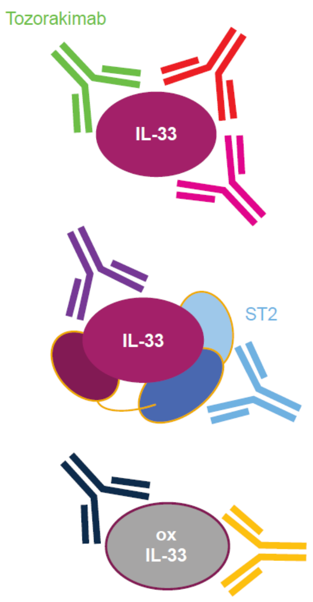

IL-33, interleukin-33; ox, oxidized; ST2, interleukin 1 receptor-like 1.

Figure S14. Assessment of urine inflammatory biomarkers in patients with DKD.

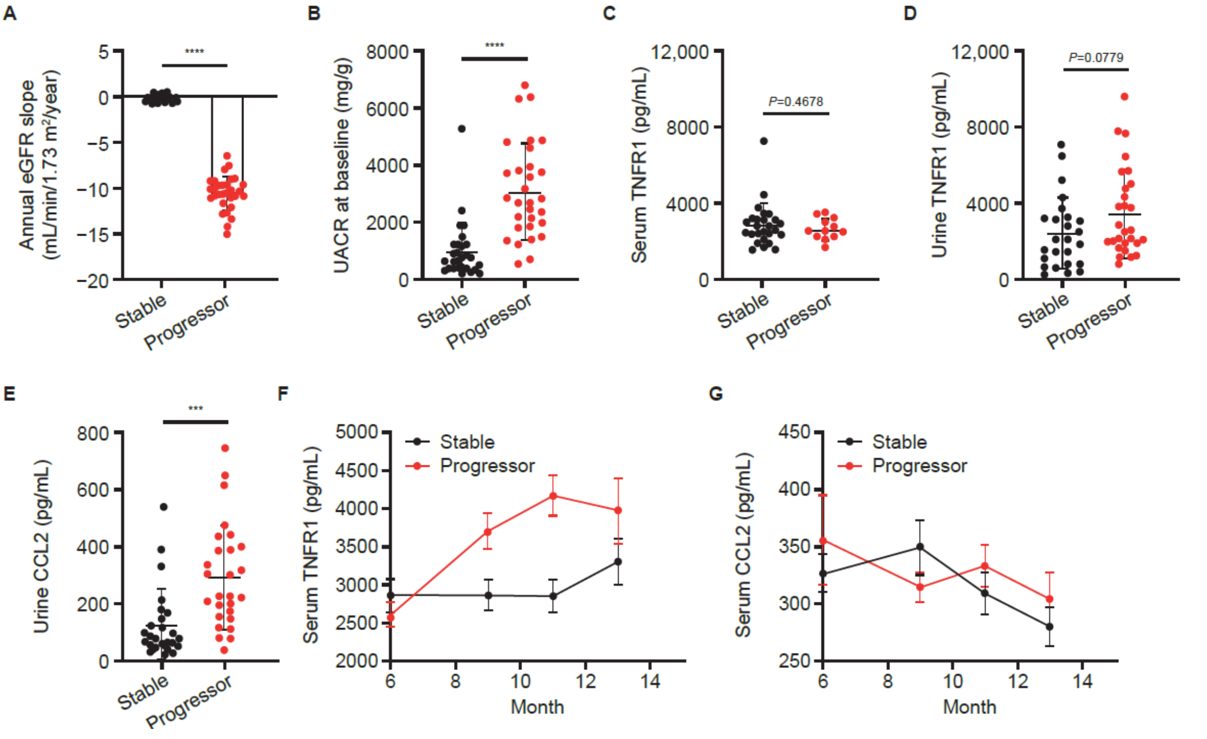

CCL2, chemokine ligand 2; DKD, diabetic kidney disease; eGFR, estimated glomerular filtration rate; TNFR1, tumor necrosis factor receptor 1; UACR, urinary albumin-to-creatinine ratio.

Figure S15. Distribution of baseline hsCRP.

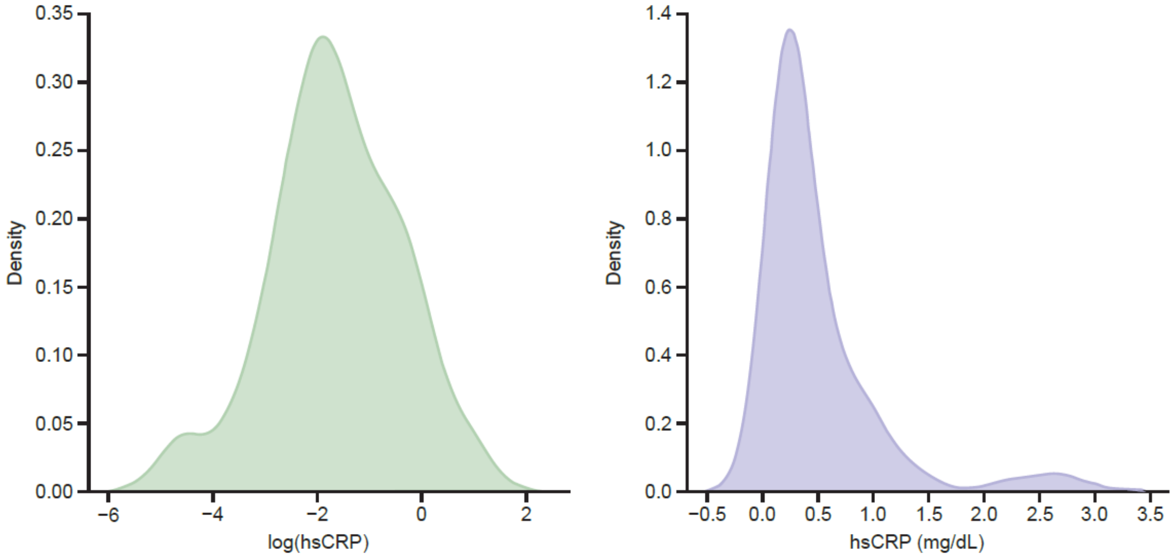

hsCRP, high-sensitivity C-reactive protein.

Figure S16. Distribution of baseline eosinophils.

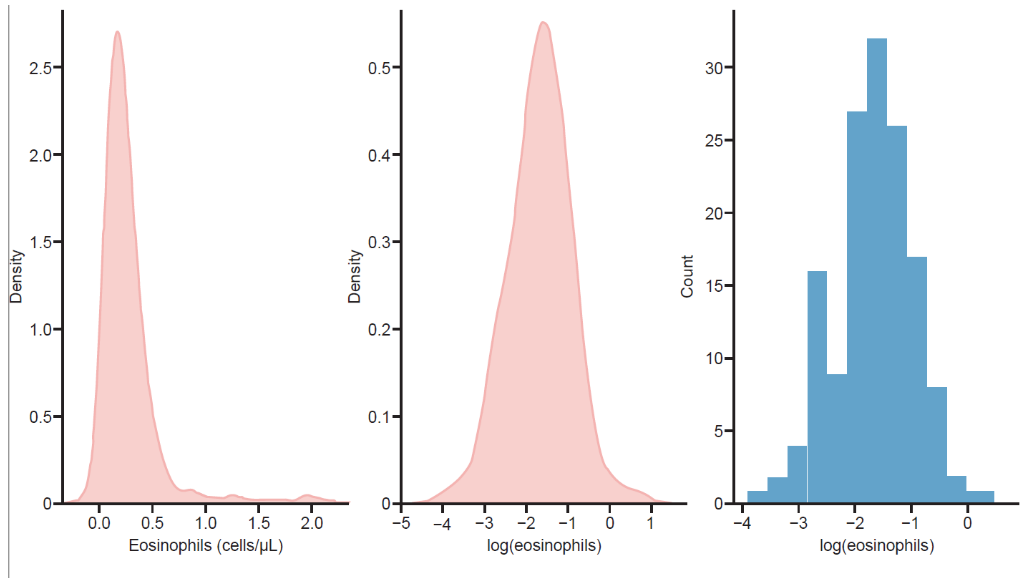

Figure S17. Histogram of baseline **(A)** CCL2, **(B)** TNFR1, and **(C)** TNFR2.

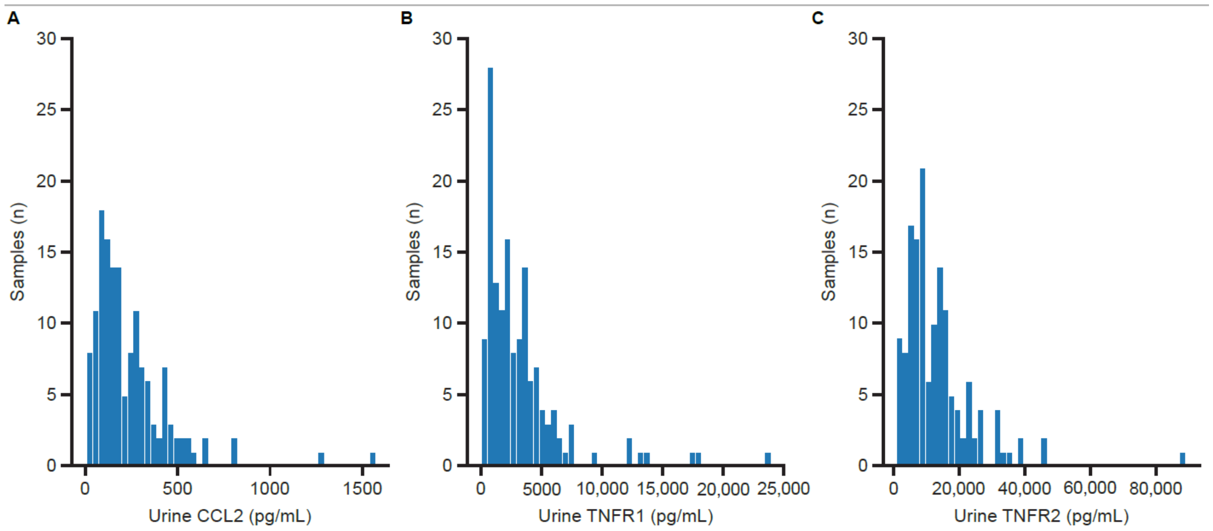

CCL2, chemokine ligand 2; TNFR1, tumor necrosis factor receptor 1; TNFR2, tumor necrosis factor receptor 2.

Figure S18. Distribution of baseline **(A)** CCL2, **(B)** TNFR1, and **(C)** TNFR2.

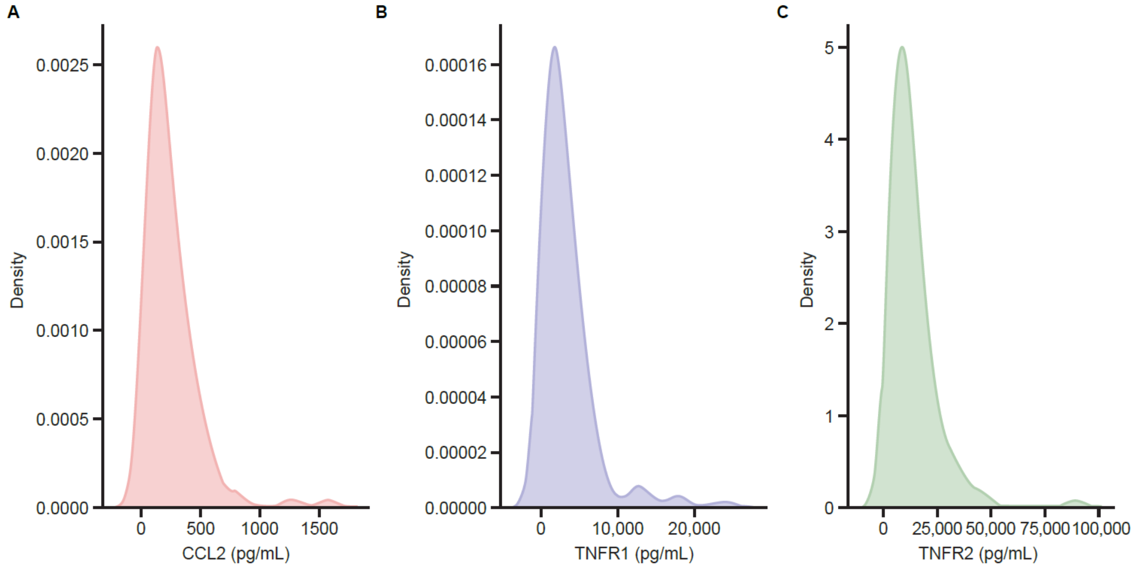

CCL2, chemokine ligand 2; TNFR1, tumor necrosis factor receptor 1; TNFR2, tumor necrosis factor receptor 2.

Figure S19. Histogram of log-transformed baseline **(A)** CCL2, **(B)** TNFR1, and **(C)** TNFR2.

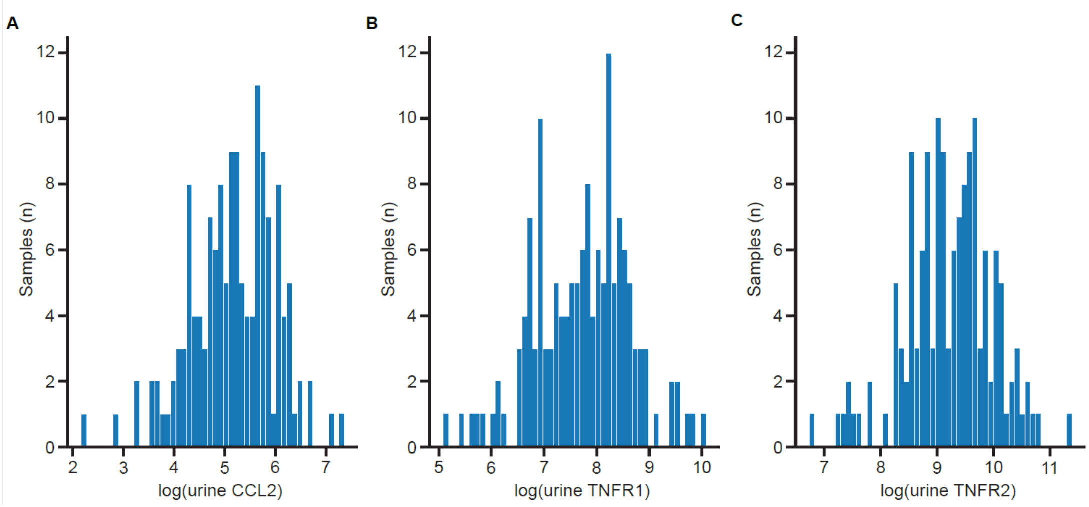

CCL2, chemokine ligand 2; TNFR1, tumor necrosis factor receptor 1; TNFR2, tumor necrosis factor receptor 2.

Figure S20. Distribution of baseline log-transformed **(A)** CCL2, **(B)** TNFR1, and **(C)** TNFR2.

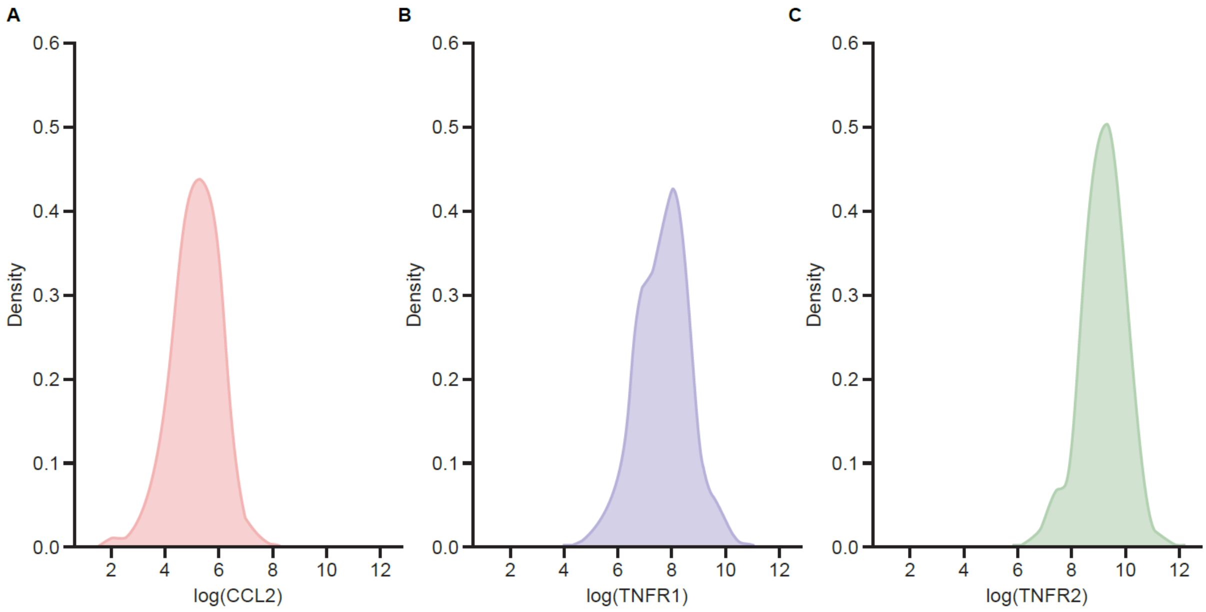

CCL2, chemokine ligand 2; TNFR1, tumor necrosis factor receptor 1; TNFR2, tumor necrosis factor receptor 2.

Figure S21. Distribution of baseline UACR.

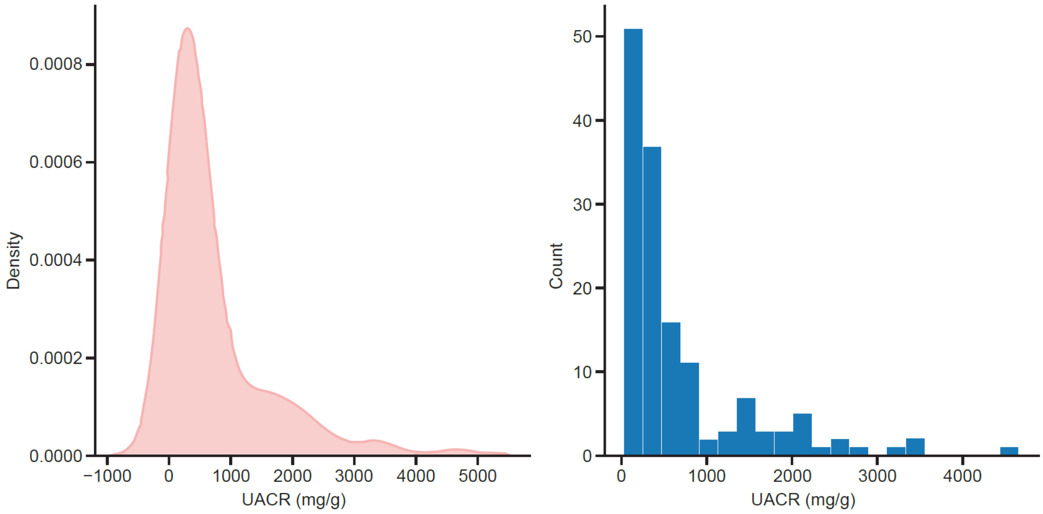

UACR, urinary albumin-to-creatinine ratio.

Figure S22. Distribution of log-transformed baseline UACR.

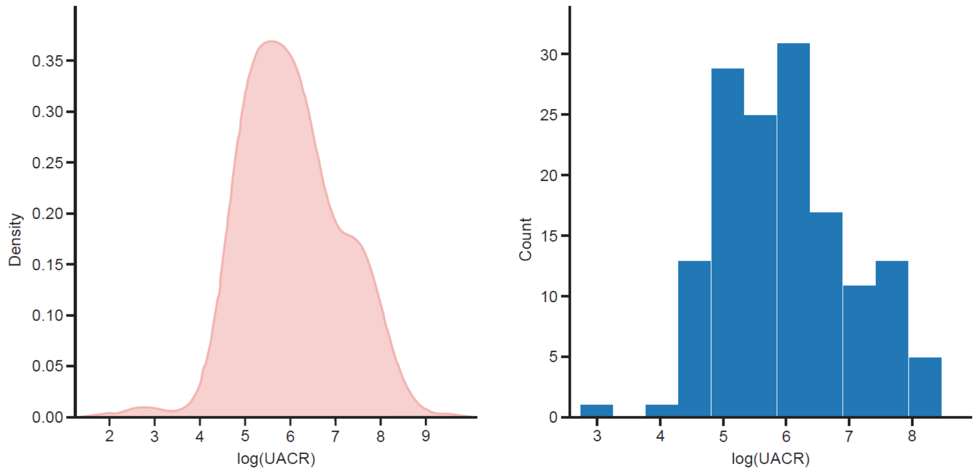

UACR, urinary albumin-to-creatinine ratio.

Figure S23. Correlation of CCL2, TNFR1, and TNFR2 with UACR.

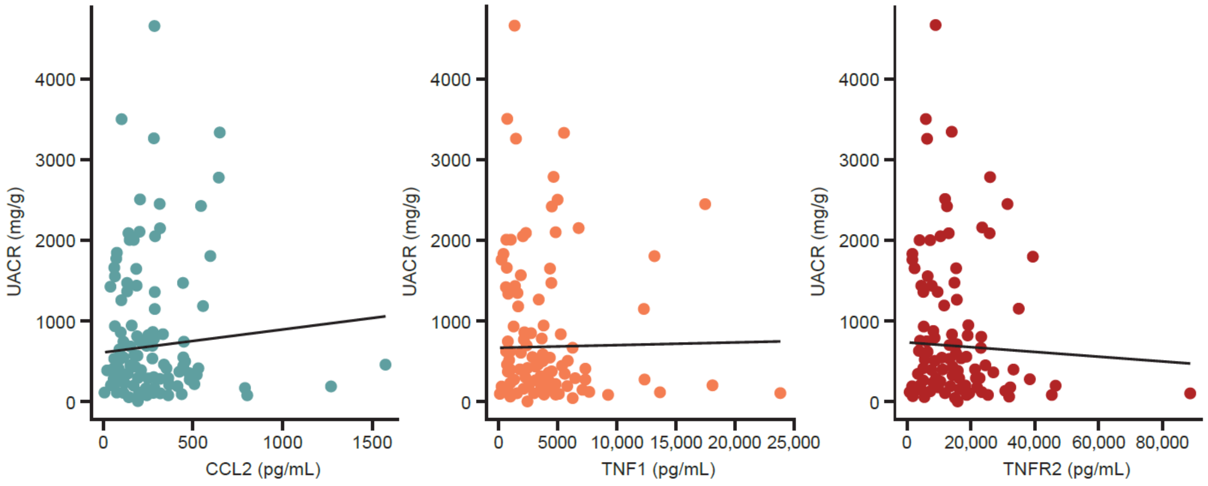

Correlation	MCP1	TNFR1	TNFR2
UACR	0.0759759	0.0142837	-0.0366559

CCL2, chemokine ligand 2; TNFR1, tumor necrosis factor receptor 1; TNFR2, tumor necrosis factor receptor 2; UACR, urinary albumin-to-creatinine ratio.

SUPPLEMENTAL REFERENCES

S1. Demidenko E: Three endpoints of in vivo tumour radiobiology and their statistical estimation. Int J Radiat Biol 86**:** 164–173, 2010 10.3109/09553000903419304
S2. Laajala TD, Corander J, Saarinen NM, Makela K, Savolainen S, Suominen MI, et al.: Improved statistical modeling of tumor growth and treatment effect in preclinical animal studies with highly heterogeneous responses in vivo. Clin Cancer Res 18**:** 4385–4396, 2012 10.1158/1078-0432.CCR-11-3215
S3. Packham DK, Wolfe R, Reutens AT, Berl T, Heerspink HL, Rohde R, et al.: Sulodexide fails to demonstrate renoprotection in overt type 2 diabetic nephropathy. J Am Soc Nephrol 23**:** 123–130, 2012 10.1681/ASN.2011040378
S4. Effect of Sulodexide in Overt Diabetic Nephropathy. https://ClinicalTrials.gov/show/NCT00130312,
S5. Petrykiv SI, Laverman GD, de Zeeuw D, Heerspink HJL: The albuminuria-lowering response to dapagliflozin is variable and reproducible among individual patients. Diabetes Obes Metab 19**:** 1363–1370, 2017 10.1111/dom.12936
S6. IMPROVE Intervention Trial Implementing Non-invasive Circulating Tumor DNA Analysis to Optimize the Operative and Postoperative Treatment for Patients With Colorectal Cancer. https://ClinicalTrials.gov/show/NCT03748680,
S7. Intensive Uric Acid Lowering With Verinurad and Febuxostat in Patients With Albuminuria. https://ClinicalTrials.gov/show/NCT03118739,
S8. Stack AG, Dronamraju N, Parkinson J, Johansson S, Johnsson E, Erlandsson F, et al.: Effect of Intensive Urate Lowering With Combined Verinurad and Febuxostat on Albuminuria in Patients With Type 2 Diabetes: A Randomized Trial. Am J Kidney Dis 77**:** 481–489, 2021 10.1053/j.ajkd.2020.09.009
S9. Woroniecka KI, Park AS, Mohtat D, Thomas DB, Pullman JM, Susztak K: Transcriptome analysis of human diabetic kidney disease. Diabetes 60**:** 2354–2369, 2011 10.2337/db10-1181
S10. Wilson PC, Wu H, Kirita Y, Uchimura K, Ledru N, Rennke HG, Welling PA, Waikar SS, Humphreys BD: The single-cell transcriptomic landscape of early human diabetic nephropathy. PNAS 116 (39) 19619-19625, 2019, 10.1073/pnas.1908706116

CONSORT STATEMENT

(see CONSORT checklist on the next page)

CONSORT 2010 checklist of information to include when reporting a randomised trial*

Section/Topic	Item No	Checklist item	Reported on page No
**Title and abstract**			
	1a	Identification as a randomised trial in the title	Abstract
	1b	Structured summary of trial design, methods, results, and conclusions (for specific guidance see CONSORT for abstracts)	Supplement
**Introduction**			
Background and objectives	2a	Scientific background and explanation of rationale	7-8 / 17-19
	2b	Specific objectives or hypotheses	17-19 / Supplement
**Methods**			
Trial design	3a	Description of trial design (such as parallel, factorial) including allocation ratio	Supplement
	3b	Important changes to methods after trial commencement (such as eligibility criteria), with reasons	Not applicable
Participants	4a	Eligibility criteria for participants	Supplement
	4b	Settings and locations where the data were collected	Supplement
Interventions	5	The interventions for each group with sufficient details to allow replication, including how and when they were actually administered	Supplement
Outcomes	6a	Completely defined pre-specified primary and secondary outcome measures, including how and when they were assessed	Supplement
	6b	Any changes to trial outcomes after the trial commenced, with reasons	Not applicable
Sample size	7a	How sample size was determined	17-19 / Supplement
	7b	When applicable, explanation of any interim analyses and stopping guidelines	Not applicable
Randomisation:			
Sequence generation	8a	Method used to generate the random allocation sequence	Supplement
	8b	Type of randomisation; details of any restriction (such as blocking and block size)	Not applicable
Allocation concealment mechanism	9	Mechanism used to implement the random allocation sequence (such as sequentially numbered containers), describing any steps taken to conceal the sequence until interventions were assigned	Supplement
Implementation	10	Who generated the random allocation sequence, who enrolled participants, and who assigned participants to interventions	Supplement

Blinding	11a	If done, who was blinded after assignment to interventions (for example, participants, care providers, those assessing outcomes) and how	Supplement
	11b	If relevant, description of the similarity of interventions	Not applicable
Statistical methods	12a	Statistical methods used to compare groups for primary and secondary outcomes	Supplement
	12b	Methods for additional analyses, such as subgroup analyses and adjusted analyses	Supplement
**Results**			
Participant flow (a diagram is strongly recommended)	13a	For each group, the numbers of participants who were randomly assigned, received intended treatment, and were analysed for the primary outcome	Analysis ongoing
	13b	For each group, losses and exclusions after randomisation, together with reasons	Analysis ongoing
Recruitment	14a	Dates defining the periods of recruitment and follow-up	Supplement
	14b	Why the trial ended or was stopped	Not applicable
Baseline data	15	A table showing baseline demographic and clinical characteristics for each group	33
Numbers analysed	16	For each group, number of participants (denominator) included in each analysis and whether the analysis was by original assigned groups	35-36 / Supplement
Outcomes and estimation	17a	For each primary and secondary outcome, results for each group, and the estimated effect size and its precision (such as 95% confidence interval)	Analysis ongoing
	17b	For binary outcomes, presentation of both absolute and relative effect sizes is recommended	Not applicable
Ancillary analyses	18	Results of any other analyses performed, including subgroup analyses and adjusted analyses, distinguishing pre-specified from exploratory	Analysis ongoing
Harms	19	All important harms or unintended effects in each group (for specific guidance see CONSORT for harms)	Analysis ongoing
**Discussion**			
Limitations	20	Trial limitations, addressing sources of potential bias, imprecision, and, if relevant, multiplicity of analyses	Not applicable
Generalisability	21	Generalisability (external validity, applicability) of the trial findings	20-22
Interpretation	22	Interpretation consistent with results, balancing benefits and harms, and considering other relevant evidence	Analysis ongoing
**Other information**			
Registration	23	Registration number and name of trial registry	NCT04170543
Protocol	24	Where the full trial protocol can be accessed, if available	Will become available
Funding	25	Sources of funding and other support (such as supply of drugs), role of funders	AstraZeneca

Citation: Schulz KF, Altman DG, Moher D, for the CONSORT Group. CONSORT 2010 Statement: updated guidelines for reporting parallel group randomised trials. BMC Medicine. 2010;8:18.
© 2010 Schulz et al. This is an Open Access article distributed under the terms of the Creative Commons Attribution License (http://creativecommons.org/licenses/by/2.0), which permits unrestricted use, distribution, and reproduction in any medium, provided the original work is properly cited.

*We strongly recommend reading this statement in conjunction with the CONSORT 2010 Explanation and Elaboration for important clarifications on all the items. If relevant, we also recommend reading CONSORT extensions for cluster randomised trials, non-inferiority and equivalence trials, non-pharmacological treatments, herbal interventions, and pragmatic trials. Additional extensions are forthcoming: for those and for up-to-date references relevant to this checklist, see www.consort-statement.org.
